# The 13th International Podocyte Conference

**DOI:** 10.1159/000525410

**Published:** 2022-06-17

**Authors:** Silke R. Brix, Durga A.K. Kanigicherla, Dean Wallace, Tejas Desai, Rachel Lennon

The podocyte is a vital component of the glomerular filtration barrier, and its dysfunction is key in many glomerular diseases. The 13^th^ International Podocyte conference was held in Manchester, United Kingdom, from July 27^th^ to 31^st^, 2021. This biannual conference has brought together clinicians, clinician-scientists and basic science researchers from across the world for over two decades to present data on podocyte biology and pathophysiology. Over the years, the meeting has developed into a key networking event for the community, and it provides an important opportunity to discuss podocyte function and to relate dysfunction to the broader spectrum of glomerular diseases. The 2021 meeting incorporated both patient and early career researcher days and had the overall aim of combining basic science with translational research and to showcase progress in the field towards novel therapies for glomerular diseases.

The meeting had 490 registrations with 379 participants for the main event, and 301 participating in the pre-meeting. 196 patients, families, researchers and industry representatives registered for the patient day. The pre-meeting focused on the early career researchers discussing disease models, *Omics* and there was a panel discussion on ‘How to become a successful podocyte researcher’. The main meeting was deliberately focused on bridging basic and translational research. The three keynote talks covered kidney organoids, a systems approach to adhesion signalling and single cell-RNA sequencing. The three-day, main meeting covered 12 themes: 1) Podocyte cell-cell and cell-matrix signalling, 2) Genetics from diagnosis to therapy, 3) Understanding basement membranes, 4) Podocyte pathology, 5) Glomerular cell crosstalk, 6) Immune mediated glomerular diseases, 7) Experimental models, 8) Drug development, 9) Podocytes in multisystem regulation and disease, 10) Next generation phenotyping, 11) Identifying new disease pathways and 12) The new therapy horizon. Each session incorporated an oral abstract presentation and importantly allowed time for expert panel discussions at the end of each session. A total of 86 abstracts were presented in the following categories: 1) Cellular crosstalk, 2) Circulating factors and disease biomarkers, 3) Disease models, 4) Drug development, 5) Genetic screening in glomerular diseases, 6) Immune mediated glomerular diseases, 7) Metabolic pathways, 8) Podocyte pathology, 9) Podocyte signalling pathways, 10) Systems biology, and the 11) Cell matrix interface. A complete catalogue with all abstracts is listed below.

Originally, the conference was planned for 2020. The emerging COVID-19 pandemic resulted in the organisers postponing the event to 2021. Due to the ongoing uncertainties of travel restrictions and the risk of contagion, a hybrid format was agreed. This allowed the combination of a face-to-face (F2F) event in Manchester with a virtual component for remote attendees. Attending virtually was quick, efficient, and safe, and from an environmental perspective minimised the carbon footprint of the conference. Different time zones presented challenges to virtual participants aiming to view all sessions live. The on-demand platform allowed participants to view sessions when convenient and also hosted longer pre-recorded talks that speakers prepared in advance. One of the biggest challenges was to continually engage a large virtual audience and keep the interaction with in-person speakers vibrant and engaging. Pivotal to this was the chat room activity and the Q+A platform *Slido* that facilitated a lively online discussion as well as a speedy transfer of questions into the live session. The feedback was extremely positive but ‘nothing replaces F2F’. F2F poster presentations for example would certainly have resulted in more personal and in-depth discussions. Personal connections and networking were more difficult online. Addressing the expense-barrier to science, registration fees were waived for low- and middle-income countries and students from all countries to improve collaborations and to minimise knowledge gaps.

Central to the strategy of hybrid content delivery was the social media strategy of #Podocyte21. Primarily, this was achieved through the Twitter social media platform and the official conference account @podocyte21. Given the earlier planned date of 2020, the account had an early start in stimulating interest and enthusiasm for the meeting within the international podocyte community. Prior to the meeting, ‘Podofact Friday’ ran weekly with key podocyte biology educational facts to whet the appetite for the scientific meeting to come. During the main meeting, a total of 245 tweets with original content were authored by 140 different individuals, all of which contained picture/slide content and 10 of which included conference video content - highlighting the free availability of high-quality educational resources for those not registered at the conference.

Of the total authors, 33/140 were registered conference attendees or speakers highlighting that the majority of tweets and interactions originated from outside the attendee pool. 385 Retweets of original material occurred during the main meeting (bringing total tweets to 630 during the main event). A word cloud summary of the meeting (**Figure 1**.) is available alongside a knowledge transfer map demonstrating the flow of educational content via twitter between participants and the meeting (**Figure 2**).

Tweeting about the meeting continued late into November 2021 prior to handover of the account to the organisers of the 14^th^ International Podocyte Conference in Philadelphia, USA in 2023. The most popular and widely re-tweeted content was the excellent pictographic artistic summaries of the speakers and key presentation points by post-doctoral researcher and science illustrator, Alex Cagan. These detailed and concise summaries were universally praised for their scientific content and artistic flair. Parallel to the scientific submissions, attendees and their contacts were encouraged to submit their ‘PodoArt’, unique artistic works involving podocyte biology, submitted by researchers and the public. These were available as an online art-show with prizes for winners and runners up.

As F2F meetings re-emerge, hybrid meeting content will continue to provide for those not able to attend in person. In the long term, hybrid models will likely remain a significant component for many events as we learn and benefit from innovations being developed during the ongoing COVID-19 pandemic. The 13^th^ International Podocyte Conference - Manchester and Online - was a successful meeting, bridging not only basic and translational science but the traditional format of conference with the new virtual way of presentation and networking. The podocyte community adapted rapidly to this novel meeting format and as such reflected the adaptive and versatile nature of the glomerular podocyte.

## Funding

The International Organising Committee of the Podocyte Conference and the authors of this article acknowledge the support of NephCure Kidney International for assistance with conference planning, advertising, fundraising and delivery. The meeting itself was supported with funding from: Travere Therapeutics, Astrazeneca, Goldfinch Bio, Nipoka, Chinook, Sanofi-Aventis, Novartis, Caliditas Therapeutics, University of Cologne, Wellcome Trust, Kidney Research UK, Kidneys for Life.

## Figures and Tables

**Figure 1:**
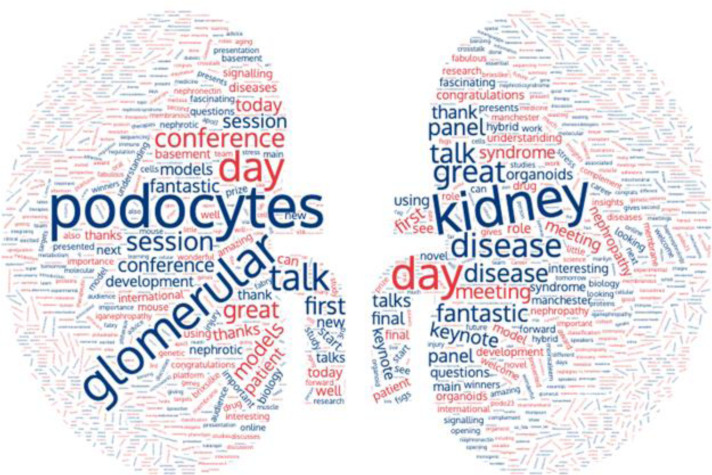
Social media word cloud summary of #podocyte21 with the most frequently mentioned keywords weighted in terms of font size.

**Figure 2:**
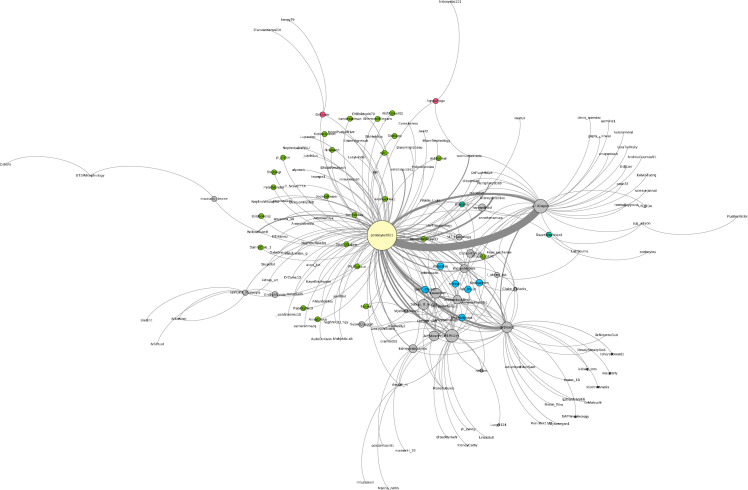
Social media knowledge transfer map with individual influencers represented as nodes and connections representing author interactions.

