# Supplementary material for: The 13th International Podocyte Conference
Source: Glomerular Dis. 2022 Jun 17;2(Suppl 1):1–78. doi: 10.1159/000525410 (PMC9672903; doi:10.1159/000525410)
Supplement: Related texts

# Abstract: Mineralocorticoid receptor drives parietal epithelial cell activation and glomerular injury during extracapillary diseases.

**Authors:** Dr Hélène Lazareth, Dr Olivia Lenoir, Dr Florian Garo, Dr Sheerazed Boulkroun, Ms Angélique Rocha, Ms Isabelle Giscos-Douriez, Dr Léa Guyonnet, Pr. Marcus J. Moeller, Dr Carole Hénique, Dr Maria-Christina Zennaro, Dr Pierre-Louis Tharaux

**Affiliations:** ^1^Inserm, Paris, France; ^2^Université de Paris, Paris, France; ^3^Institut Curie, Paris, France; ^4^Rheinisch- Westfälische Technische Hochschule (RWTH) University Hospital Aachen, Aachen, Germany; ^5^Inserm, Créteil, France; ^6^University Paris-Est-Créteil (UPEC), Créteil, France; ^7^Assistance Publique-Hôpitaux de Paris, Paris, France

## Background

MR is strongly expressed in endothelial cells, glomerular mesangial cells, podocytes, and distal tubular cells. The role of the MR in extracapillary glomerulopathies is still elusive and mechanistically unclear.

## Methods

We examined the MR expression in PECs in human kidneys. We generated mice specifically lacking MR (Pec Cre Nr3c2 lox/lox) in PECs. We challenged these mice with the uninephrectomy and DOCA-salt (UNx-DOCA-salt) model of focal segmental glomerulosclerosis (FSGS), and the nephrotoxic serum (NTS) model of crescentic glomerulonephritis (CGN).

## Results

First, we found constitutive expression of the MR in PECS from normal human kidneys that was increased in extracapillary lesions of patients diagnosed with FSGS or CGN. Pec Cre Nr3c2 lox/lox displayed significantly fewer CD44 abundance than UNx-DOCA-salt control animals. When challenged with NTS, Pec Cre Nr3c2 lox/lox developed less albuminuria and milder kidney failure than Pec Cre Nr3c2 wt/wt counterparts. Glomerular injury and PEC activation markers (CD44, CD9, and fibronectin) were significantly blunted in Pec Cre Nr3c2 lox/lox mice. Next, oral administration of eplerenone for 14 days after NTS significantly decreased proteinuria and BUN, reduced expression of CD44 on PECs and number of crescents.

## Conclusion

These results indicate the role of MR in PEC activation in the UNx-DOCA-salt model of FSGS and during CGN along with the recently discovered CD9/EGFR/PDGFR pathway, supporting the idea that the PEC phenotype switch is not a bystander event but plays a targetable active pathogenic role. MR modulation may be a new therapeutic option for the management of extracapillary diseases.

# Abstract: 1 The Role of VEGFR3 Signalling in Glomerular Capillary Loop Development

**Authors:** Dr Michael Donnan, Dr Tuncer Onay, Dr Rizaldy Scott, Dr Dilip Deb, Dr Jean-Léon Thomas, Dr Susan Quaggin

**Affiliations:** ^1^Northwestern University Feinberg School of Medicine, Chicago, USA; ^2^Yale School of Medicine, New Haven, USA

Dysregulation of Vascular Endothelial Growth Factor Receptor 3 (VEGFR3), known primarily for its role in lymphangiogenesis, is causally linked to the development of kidney diseases making it a potential target for new therapeutics. However, the role of VEGFR3 signalling in kidney development, how it influences kidney disease, and the vascular beds involved remains uncertain.

We performed a detailed expression profile of VEGFR3 in the developing mouse kidney. We generated a new transgenic mouse model to investigate the role of Vegfr3 in the kidney vasculature (Vegfr3Flx). Conditional and cell-specific excision of the floxed allele was performed using the Rosa-rtTA-TetOCre, Cdh5-Cre/ERT2, and Prox1-Cre/ERT2 driver strains to evaluate global, pan-endothelial, and lymphatic endothelial cell deletion of Vegfr3 respectively. Mice underwent a detailed phenotypic evaluation and kidney sections were processed for histology.

VEGFR3 undergoes dynamic expression through development in several fenestrated blood microvascular beds of the kidney including glomerular endothelial cells (GECs). Constitutive deletion of Vegfr3 during mid-embryonic development resulted in reduced viability, lymphatic vascular defects, and a reduction in kidney size. Additionally, deletion of Vegfr3 at embryonic day 11.5 resulted in marked disruption of glomerular development with cavernous capillary malformations. Immunofluorescence and electron microscopy revealed glomerular structures surrounded by simplified podocytes, abnormal attachment of endothelial cells with reduced fenestrations, and poor formation of the glomerular basement membrane.

VEGFR3 is expressed in GECs and is integral to normal glomerular development. The mechanisms of VEGFR3 signalling in GEC crosstalk with podocytes will be essential to define prior to the development of therapeutics targeting this pathway.

# Abstract: 2 Shiga toxin targets the podocyte in Haemolytic Uraemic Syndrome resulting in endothelial complement dysregulation

**Authors:** Dr Emily Bowen, Dr Jenny Hurcombe, Dr Lindsay Keir, Miss Fern Barrington, Dr Louise Farmer, Dr Abigail Lay, Dr Gavin Welsh, Miss Eva Larkai, Prof Moin Saleem, Prof Richard Coward

**Affiliations:** University of Bristol, Bristol, United Kingdom

## Background

Haemolytic uraemic syndrome (HUS) is a thrombotic microangiopathy (TMA) that has a predilection to present in the kidney. It is a triad of microangiopathic haemolytic anaemia, thrombocytopenia, and acute kidney injury. In 90% of cases, HUS follows gastroenteritis secondary to infection with Shiga toxin (Stx) producing bacteria such as Escherichia coli. Stx HUS is the leading cause of acute kidney injury in children with a mortality of 5%. However, the precise pathophysiological mechanisms following Stx infection leading to TMA remain poorly understood. Here we show that the podocyte is a key initiator in Shiga toxin HUS, which could explain why the glomerulus is the prime target of systemic Shiga toxin driven infection.

## Methods

To demonstrate that the podocyte Shiga toxin receptor (Gb3) is sufficient to trigger the development of HUS, we used conditional gene targeting to engineer human Gb3 expression specifically in the podocytes of adult mice.

## Results

Following intraperitoneal Shiga toxin challenge, these mice developed thrombocytopenia, haemolytic anaemia, and uraemia with evidence of glomerular TMA on histology. Interrogation of this model revealed evidence of glomerular endothelial cell complement activation, with loss of local complement factor H protection. Furthermore, C5 inhibition was found to rescue the Shiga toxin HUS phenotype.

## Conclusions

Together, these observations provide compelling evidence for the importance of podocyte- glomerular endothelial cell crosstalk within the kidney in the development of Shiga toxin associated HUS and suggest a possible therapeutic role for complement inhibition in patients with this devastating disease.

# Abstract: 3 A central role of Endothelin A Receptor Activation in Mesangial Cell - Podocyte Crosstalk in IgA Nephropathy and Other Mesangio-Proliferative Glomerulopathies

**Authors:** Dr. Jennifer Cox, Mr. Marvin Gunawan, Ms. Joyce Wu, Mr. Jeff Lester, Mr. Charles Nieh, Olsonxy2Dr. Eric, Dr. Jay Kuo, Dr. Andrew King

**Affiliations:** Chinook Therapeutics, Vancouver, Canada

## Introduction

Mesangial cell (MC) activation by IgA-immune complexes is the initiating intra-renal event in the pathogenesis of IgAN. Subsequent MC-podocyte crosstalk results in proteinuria, the strongest predictor of progression. However, the molecular mechanisms responsible have not been well defined. The objective of these studies was to determine the role of the ETA receptor in mesangial cell activation and proteinuria in IgAN and other mesangio-proliferative glomerulopathies, using the potent and selective ETA antagonist atrasentan.

## Methods

The effect of atrasentan was assessed on ET-1 or IgA-immune complex activation MCs and in the gddY and anti-Thy-1.1 models.

## Results

ET-1 directly stimulated proliferation and IL-6 secretion in primary human MCs, which was blocked by atrasentan in a concentration-dependent manner. RNA sequencing and gene set enrichment analysis revealed hallmarks of MC activation with ET-1 treatment including up-regulation of cell proliferation, pro-inflammatory and pro-fibrotic networks, which were blocked by atrasentan. MCs exposed to IgA-immune complexes purified from the serum of IgAN patients demonstrated hyper- proliferation relative to complexes from healthy controls, which was significantly attenuated by atrasentan. In the gddY mouse model of spontaneous IgAN, atrasentan rapidly and significantly reduced albuminuria and down-regulated intra-renal proliferative, inflammatory, and fibrotic transcriptional networks. In the rat anti-Thy-1.1 antibody induced model of mesangio-proliferative glomerulopathy, atrasentan attenuated glomerular injury histologically, including mesangial hypercellularity and matrix expansion, segmental mesangiolysis and glomerulosclerosis. In addition, atrasentan significantly reduced proteinuria and tubulointerstitial injury score.

## Conclusion

These studies suggest an important role of the ETA receptor in MC activation and subsequent proteinuria in IgAN and other mesangio-proliferative glomerulopathies.

# Abstract: 1 Investigation of the glomerular basement membrane and penetration of microparticles though the glomerular filtration barrier in different nephronectin knockout models

**Authors:** PD Dr. Janina Müller-Deile, Dr. Nina Sopel, Ms Alexandra Ohs, Prof. Dr. Mario Schiffer

**Affiliations:** Friedrich-Alexander-Universität Erlangen-Nümberg, Erlangen, Germany

## Background

Nephronectin (NPNT) is an extracellular matrix protein downregulated by TGF-β or miR- 378a in podocytes. Knockdown (KD) of npnt leads to proteinuria, podocyte effacement and thickening of the glomerular basement membrane (GBM) in zebrafish larvae. Methods: We characterized the phenotype of npnt KD in more detail by using serial block face scanning electron microscopy in zebrafish. Penetration of albumin size nanoparticles and IgG size nanoparticles through the GFB was analyzed in control as well as Npnt KD models in zebrafish larvae by electron microscopy. We further investigated the role of Npnt in two mouse modes where Npnt was specifically knocked out in podocyte progenitor cells (Npnt fl/fl;Six2-cre mice) or podocytes (Npnt fl/fl;Nphs2-cre mice).

## Results

In addition to the previously described podocyte-derived miR-378a-3p, we identified glomerular endothelial cell-derived miR-192-5p as a regulator of NPNT in podocytes. Overexpression of miR-192-5p in zebrafish larvae induced edema, proteinuria and GBM thickening similar to the phenotype after morpholino-based npnt KD. Albumin size nanoparticles passed the GFB and were taken up by microvilli of zebrafish tubuli in CTRL and npnt KD zebrafish. In contrast, IgG size nanoparticels accumulated at the blood side of glomerular endothelial cells in CTRL zebrafish but were distributed throughout the whole GFB and also reached the tubuli npnt KD zebrafish. The unique thickened GBM was further confirmed Npnt fl/fl;Six2-cre and Npnt fl/fl;Nphs2-cre mice.

## Conclusion

The results confirm the role of podocyte npnt for proper GBM function and suggests its regulation by podocyte- and glomerular endothelial cell-derived miRs.

# Abstract: 2 Identification of an altered matrix signature in kidney ageing and disease

**Authors:** Dr Michael Randles, Dr Franziska Lausecker, Dr Qingyang Kong, Dr Hani Suleiman, Dr Graeme Reid, Dr Maria Kolatsi-Joannou, Dr Bernard Davenport, Dr Pinyuan Tian, Dr Sara Falcone, Dr Paul Potter, Dr Tom Van Agtmael, Professor Jill Norman, Dr David Long, Professor Martin Humphries, Professor Jeffrey Miner, Professor Rachel Lennon

**Affiliations:** ^1^University of Chester, Chester, United Kingdom; ^2^University of Manchester, Manchester, United Kingdom; ^3^University College London, London, United Kingdom; ^4^Washington University School of Medicine, Saint Louis, USA; ^5^Manchester Royal Infirmary, Manchester, United Kingdom; ^6^University of Oxford, Oxford, United Kingdom; ^7^Oxford Brookes, Oxford, United Kingdom; ^8^University of Glasgow, Glasgow, United Kingdom

## Background

Accumulation of extracellular matrix in organs and tissues is a feature of both ageing and disease. In the kidney, glomerulosclerosis and tubulointerstitial fibrosis accompany the decline in organ function, which is irretrievable with current therapies leading to inevitable kidney failure. Whilst histological and ultrastructural patterns of excess matrix form the basis of human disease classifications, the comprehensive molecular resolution of abnormal matrix is lacking.

## Methods

Using mass spectrometry-based proteomics we resolved matrix composition over age in mouse models of kidney disease. We compared the changes in mice with a global characterization of human kidney matrix during ageing and to existing kidney disease datasets to identify common molecular features.

## Results

We found that ultrastructural changes in basement membranes are associated with altered cell adhesion and metabolic processes and with distinct matrix proteomes during ageing and kidney disease progression in mice. Within the altered matrix, we observed a reduction in basement membrane components (laminins, type IV collagen, type XVIII collagen) and a corresponding increase in interstitial matrix proteins (collagens I, III, VI, XV, fibrinogens and nephronectin) and this pattern was also seen in human kidney ageing. Indeed, this signature of matrix proteins was consistently modulated across all age and disease comparisons and the increase in interstitial matrix was also observed in human kidney disease datasets.

## Conclusions

This study provides deep molecular resolution of matrix accumulation in kidney ageing and disease and identifies a common signature of proteins that provides insight into mechanisms of response to kidney injury and repair.

# Abstract: 1 Identification of new force regulators in the kidney glomerulus

**Authors:** Dr Franziska Lausecker, Dr Michael Randles, Dr Craig Lawless, Dr Anna Li, Dr Ben Dickie, Dr Bernard Davenport, Prof Rachel Lennon

**Affiliations:** ^1^Wellcome Centre for Cell-Matrix Research, Division of Cell-Matrix Biology and Regenerative Medicine, School of Biological Sciences, Faculty of Biology Medicine and Health, The University of Manchester, Manchester Academic Health Science Centre, Manchester, United Kingdom; ^2^University of Chester, Chester, United Kingdom; ^3^University of Manchester, Manchester, United Kingdom; ^4^Department of Paediatric Nephrology, Royal Manchester Children's Hospital, Manchester University Hospitals NHS Foundation Trust, Manchester Academic Health Science Centre, Manchester, United Kingdom

## Background

Hypertension contributes to glomerular disease progression, often leading to end-stage kidney disease. As a result, Renin-Angiotensin-Aldosterone Inhibitors are used for treatment of glomerular diseases. Even though blood pressure is targeted for therapy, the underlying molecular mechanism of force regulation in glomerular health and disease remains to be elucidated.

## Methods

We performed unilateral nephrectomies (UNx) in wild-type and Alport mice to investigate acute and long-term cellular and matrix responses to increased mechanical load. We measured the glomerular response using electron microscopy (EM), magnetic resonance imaging (MRI) and mass spectrometry (MS)-based proteomics.

## Results

MRI demonstrated increased blood flow post-surgery in wild type mice and nephrectomy- induced hypertrophy. Whilst kidney function and microanatomy was preserved, we found thickening of the glomerular basement membrane and the formation of podocyte invasions. MS of glomeruli revealed moderate changes in the glomerular matrix of the remaining kidney following UNx, including downregulation of Col1a1 and upregulation of Adamtsl4. Significant protein changes were observed in the cellular components, including downregulation of Myo1e and Ptk2, suggesting an adaptive cellular response to mechanical load.

Whilst in wild-type mice albumin-creatinine ratios were preserved, a rapid decline in kidney function was observed in Alport mice post-surgery. Moreover, EM demonstrated disorganisation of the glomerular filtration barrier. MS analysis revealed opposing regulation of proteins in the Alport model, indicating a protective role for specific mechanoregulators in wild-type mice.

## Conclusions

This study provides deep molecular resolution of glomerular response to mechanical load. We identify new force-responsive proteins, which could be targeted in glomerular disease.

# Abstract: 2 Investigating basement membrane assembly in human kidney organoids

**Authors:** Dr Pinyuan Tian, Dr Mychel Morais, Dr Craig Lawless, Dr Syed Murtaza-Baker, Miss Louise Hopkinson, Dr Steven Woods, Dr Aleksandr Mironov, Prof David Long, Prof Daniel Gale, Prof Telma Zorn, Prof Susan Kimber, Prof Roy Zent, Prof Rachel Lennon

**Affiliations:** ^1^University of Manchester, Manchester, United Kingdom; ^2^University College London, London, United Kingdom; ^3^University of Sao Paulo, Sao Paulo, Brazil; ^4^Vanderbilt University Medical Center, Nashville, USA

## Background

Basement membranes (BMs) are condensed sheets of extracellular beneath continuous layers of cells matrix composed primarily of type IV collagen and laminins. In the kidney, the glomerular basement membrane (GBM) between the podocytes and the glomerular endothelial cells is a critical component of the glomerular filtration barrier. Previous studies revealed that the GBM composition undergoes profound changes throughout kidney development, but little is known about mechanisms that regulate BM assembly and isoform transitions due to lack of proper models. Human pluripotent stem cells (hPSCs)-derived kidney organoids have great potential for investigating kidney development and disease modelling. Methods: Human induced pluripotent stem cells (iPSCs) were derived from peripheral blood mononuclear cells (PBMCs) of a healthy individual and were differentiated to kidney organoids following a modified protocol from Takasato et al. in 2015.

## Results

Whole mount immunofluorescence demonstrated that kidney organoids differentiate into glomerular structures containing podocytes (WT1+, NPHS1+), proximal tubule (LTL+), distal tubule (ECAD+), and endothelial cells (CD31+). Notably, immunostaining of the key BM markers showed that organoids form BM during differentiation, and more importantly, recapitulate a sequence of assembly events with initial deposition of laminin followed by incorporation of type IV collagen, nidogen and perlecan. Single-cell RNA sequencing analysis from day 25 kidney organoids (Combes et al., 2019) confirmed NPHS2+/PODXL+ podocytes were the main source of COL4A3, COL4A4.

## Conclusions

These findings fit with the current understanding of kidney development *in vivo* and demonstrate that kidney organoids can efficiently recapitulate the spatiotemporal emergence of GBM components during glomerulogenesis.

# Abstract: 3 Loss of Myosin 1e in Podocytes Leads to a Deficiency of Fibronectin Matrix and Mislocalization of Vinculin

**Authors:** Mr. Michael Garone, Mrs. Sharon Chase, Dr. Mira Krendel

**Affiliations:** Upstate Medical University, Syracuse, USA

## Background

Podocytes are renal epithelial cells attached to the extracellular matrix (ECM) layer known as the glomerular basement membrane (GBM), which is synthesized by podocytes and glomerular endothelial cells and surrounds glomerular capillaries. When podocytes or the GBM are damaged, renal filtration is compromised, often leading to the development of Chronic Kidney Disease (CKD). Mutations in an actin-dependent molecular motor protein in podocytes, myosin 1e (myo1e), have been identified as causative in certain CKD patients. Correspondingly, we have shown that the absence of myo1e in our Myo1e-knockout (KO) mice also leads to the development of CKD.

## Methods

We used conditionally immortalized podocytes to examine fibronectin expression and deposition under static culture conditions and under fluid flow.

## Results

We demonstrate that podocytes isolated from our Myo1e-KO mice exhibit lower levels of fibronectin in the ECM than myo1e heterozygous or wild type (WT) podocytes. Myo1e-KO podocytes also exhibited altered localization of the focal adhesion protein vinculin. Additionally, fibronectin mRNA expression appeared to be less upregulated in response to flow in Myo1e-KO podocytes than in the WT podocytes.

## Conclusions

The observed vinculin mislocalization and the decreased levels of extracellular fibronectin may affect podocyte adhesion to the GBM. Though the results of our flow experiments are preliminary, they may indicate that myo1e-deficient podocytes are less adaptable to mechanical stress. Ultimately, our findings suggest that the pathology observed in both mice and patients with myo1e deficiencies may be the result of an altered GBM composition.

# Abstract: 1 Soluble RARRES1 induces apoptosis of podocytes to promote progression of kidney disease

**Authors:** Dr Anqun Chen, Dr Tianjun Guan, Dr Kyung Lee, Dr John Cijiang He

**Affiliations:** ^1^Zhongshan Hospital, Xiamen University, Xiamen, China; ^2^Division of Nephrology, Department of Medicine, Icahn School of Medicine at Mount Sinai, New York, USA; ^3^Renal Section, James J. Peters VAMC, New York, USA

## Background

Podocyte loss is a major event leading to the progression of focal segmental glomerulosclerosis (FSGS) and diabetic nephropathy (DN). Here, we found that retinoic acid receptor responder protein 1 (RARRES1) contributes to the podocyte loss in FSGS and DN. Methods: We determined the role of RARRES1 in human and mouse with FSGS and DN. We investigated the mechanisms of RARRES1 in cultured human podocytes.

## Results

We found that expression of RARRES1 increased in the glomeruli of patients with FSGS and DN and correlated with the eGFR. Single-cell RNA-sequencing of the kidney showed that RARRES1 expressed highly in podocytes. Immunostaining confirmed that podocyte expression of RARRES1 increased in patients with DN and FSGS as compared to minimal change disease. Expression of RARRES1 in cultured human podocytes was upregulated by TNF-a. RNA-sequencing of podocytes with RARRES1 overexpression revealed genes enriched in apoptosis and overexpression of RARRES1 in podocytes induced apoptosis. Mechanistically, RARRES1 induced podocyte apoptosis through the cleavage into a soluble form which underwent endocytosis and then interacted with atypical protein kinase RIOK1 to activate p53. We mapped the cleavage site at 71^st^ amino acid of RARRES1 and mutation of this site abolished its apoptotic effect. In vivo, induction of RARRES1 overexpression in podocytes caused albuminuria and glomerular injury in normal mice and aggravated kidney injury in mice with DN and FSGS whereas induction of RARRES1 with 71^st^ amino acid mutation did not.

## Conclusion

Our study reveals a novel role and mechanism of RARRES1 in podocyte injury in glomerular disease.

# Abstract: 2 Serum and urine levels of Klotho as early biomarkers of diabetic nephropathy

**Authors:** Dr. Marlena Typiak, Tomasz Kulesza, Patrycja Rachubik, Prof. Agnieszka Piwkowska

**Affiliations:** ^1^Laboratory of Molecular and Cellular Nephrology, Mossakowski Medical Research Institute, Polish Academy of Sciences, Gdansk, Poland; ^2^Faculty of Chemistry, University of Gdansk, Gdansk, Poland

Diabetes mellitus (DM) affects ˜10% of a world adult population. About 40% of DM patients develop kidney dysfunction - diabetic nephropathy (DN), which in later stages often requires renal replacement therapy. Therefore, there is a great need for early DN biomarkers, which is still not met. Because the anti-aging and anti-inflammatory α-Klotho (KL) protein level was decreased in serum and urine of individuals with acute kidney injury, we analyzed its usefulness as the early biomarker of DN.

Therefore, we measured Klotho level in serum, urine (ELISA), and renal tissue (Western blot, immunohistochemistry) in male Wistar rats with streptozotocin-induced (STZ) diabetes and age- matched healthy controls. We measured mRNA (Real-time PCR) and total KL protein expression (Western blot, immunofluorescence) in human immortalized podocytes, cultured in diabetic (hyperglycemic, HG) or normoglycemic (NG) conditions, and membrane KL (mbKL) level in these cells after soluble KL (sKL) shedding to the cell medium (biotinylation, Western blot).

We found that serum sKL levels increased, whereas Klotho urine excretion, Klotho level in stained renal tissue and Klotho protein expression in glomeruli decreased in STZ *vs.* control rats. mRNA and total protein expression of KL in human podocytes was not dependent on NG/HG conditions. However, mbKL level in podocytes decreased, while sKL shedding increased in HG.

As previous studies showed no disruption in serum Klotho level in DM patients without nephropathy, and a link between low urinary KL levels and podocyte dysfunction, the revealed increased serum and decreased urinary Klotho levels could be considered as early biomarkers of DN.

# Abstract: 3 Phosphoproteome and raft proteome changes of human podocytes in response to recurrent idiopathic nephrotic syndrome plasma

**Authors:** Dr Cerina Chhuon, Mr. Berkan Savas, Dr Cécile Charrière-Bertrand, Dr Shao-Yu Zhang, Dr Vincent Audard, Dr Dil Sahali, Dr Ida Chiara Guerrera, Pr. Mario Ollero

**Affiliations:** ^1^Proteomics Platform Necker, Paris, France; ^2^INSERM U955, Créteil, France; ^3^Henri Mondor Hospital, Créteil, France

## Background

Idiopathic nephrotic syndrome (INS) comprises a group of rare glomerulopathies attributed to direct or indirect interaction of putative circulating factors with podocytes. One of the pathological manifestations of INS is the rapid recurrence of focal and segmental glomerulosclerosis (rFSGS) after renal transplant in certain individuals. To date, the nature and identity of circulating factors has not been confirmed, while the pathogenic mechanisms in the podocyte are partially understood. We aimed to study the early proximal signalling events in podocytes in response to plasma from post-transplant rFSGS patients, by a combination of phosphoproteomics and lipid raft proteomics (raftomics).

## Methods

We incubated differentiated human podocytes for 30min with 10% of first post-transplant plasma exchange fluid from either rFSGS or non-INS patients, the latter used as controls. We performed differential phosphoproteomics after phosphopeptide enrichment in a TiO2 column. In parallel, we isolated lipid rafts by a detergent-free method and performed differential proteomics by LC-MS/MS analysis.

## Results

The ensemble of results showed specific differences in protein expression and phosphorylation associated with rFSGS plasma incubation, suggesting alterations in the mTOR pathway, in autophagy, in mitochondrial metabolism, and in cytoskeleton organization. In particular, validation experiments suggested an altered phosphorylation pattern of Hsp27. Conclusions: The observed changes associated with rFSGS plasma incubation highlight some of the mechanisms involved in FSGS recurrence and could be used as specific early markers of circulating factor activity on podocytes.

# Abstract: 4 In vitro assays to demonstrate the presence of plasma-derived circulating permeability factors in the pathogenesis of focal segmental glomerulosclerosis

**Authors:** Susan Veissi, Bart Smeets, Joanna A. E. van Wijk, Floor Veltkamp, René Classens, Thea van der Velden, Annelies Jeronimus-Klaasen, William Morello, MD Giovanni Montini, Antonia Bouts, Prof. Bert van den Heuvel, Prof. Michiel F. Schreuder

**Affiliations:** ^1^Department of Pediatric Nephrology, Amalia Children's Hospital, Radboud university medical center, Radboud Institute for Molecular Life Sciences, Nijmegen, Netherlands; ^2^Department of Pathology, Radboud university medical center, Radboud Institute for Molecular Life Sciences, Nijmegen, Netherlands; ^3^Department of Pediatric Nephrology, Amsterdam University Medical Center, Amsterdam, Netherlands; ^4^Department of Pediatric Nephrology, Amsterdam UMC, University of Amsterdam, Amsterdam, Netherlands; ^5^Department of Pediatric Nephrology, Dialysis and Transplant Unit, Foundation IRCCS Cà Granda, IRCCS Ospedale Maggiore Policlinico, Milan, Netherlands

## Background

Circulating permeability factors (CPFs) causing idiopathic focal segmental glomerulosclerosis (FSGS) can lead to early recurrence of FSGS and kidney failure after transplantation. Identification of FSGS patients with CPFs is clinically important as it can predict treatment response and prognosis. Using human podocytes as a substrate, we aim to demonstrate the presence of CPFs using series of *in vitro* assays.

## Methods

Podocytes (ciPods) and endothelial cells (GMVECs) were incubated with plasma from biopsy proven FSGS patients in relapse and remission and from steroid-resistant nephrotic syndrome (SRNS), minimal change NS (MCNS), membranous nephropathy (MN), and healthy controls (hCtrls). Cell viability, podocyte actin cytoskeleton architecture, and reactive oxygen species (ROS) formation in the presence or absence of ROS scavenger, dimethylthiourea, were investigated by CCK-8 assay, immunofluorescence staining, and CM-H2DCFDA probing, respectively.

## Results

We show that plasma of patients with FSGS causes a series of events in podocytes but not in endothelial cells. These events include actin cytoskeleton rearrangement, excessive formation of ROS, and eventually also cell death. These effects were solely observed in response to plasma of relapse FSGS patients, but not in response to plasma of patients with SRNS, MCNS, MN, and hCtrls. The co-presence of dimethylthiourea, abolished these effects. Conclusions: Altogether, we provide a panel of assays to measure podocyte injury and predict the presence of CPFs in FSGS plasma, providing a new framework for monitoring CPF activity that can be used for diagnostics or disease monitoring purposes. Moreover, the inhibition of ROS formation may exert beneficial effects in FSGS patients.

# Abstract: 1 A novel Nano-luciferase transgenic reporter to measure proteinuria in zebrafish

**Authors:** Dr Richard Naylor, Mr Emmanuel Lemarie, Dr Bernard Davenport, Dr Aleksandr Mironov, Ms Emily Williams, Professor Martin Lowe, Professor Rachel Lennon

**Affiliations:** University of Manchester, Manchester, United Kingdom

## Background

Proteinuria is a key primary clinical indicator of kidney disease. In humans, measuring proteinuria is relatively simple, but in model organisms used in laboratory settings this can be more difficult. This is particularly the case for the zebrafish, which has become an important system to investigate kidney development and disease. Their aquatic environment and small urine volume makes quantitative analysis of urine difficult.

## Methods

We have used Tol2 transgenics to develop a Nanoluciferase transgenic zebrafish reporter of proteinuria. Using a luminometer, analysis of embryonic media can be assayed in a high-throughput manner. We used morpholino and CRISPR-Cas9 knockdown approaches as well as chemical treatments to demonstrate the usability of our reporter. Proteomics was used to identify the glomerular proteome in 5 day-old zebrafish.

## Results

Our proteinuria reporter is able to detect dysfunction in both the glomerulus and proximal tubules. We establish the zebrafish as a model of Alport syndrome, which is caused by mutations in the *COL4A3/COL4A4/COL4A5* genes in humans. Orthologs to these genes are expressed in the zebrafish glomerulus and caused proteinuria upon depletion. Conclusions: Our new tool for quantifying proteinuria in the zebrafish enables this system to be used to assay the effects of genetic alterations or chemical treatments on glomerular and proximal tubule function in a high-throughput manner. The viability of the zebrafish as a vertebrate model with which screening of chemicals can be performed means that this new tool has the potential to establish the zebrafish as a key model for future drug testing.

# Abstract: 2 Novel podocin mutants mistrafficking in kidney organoid models of congenital nephrotic syndrome

**Authors:** Dr Aude Dorison, Mrs Irene Ghobrial, Dr Sara E Howden, Miss Alison Graham, Dr Thomas A Forbes, Prof Melissa H Little

**Affiliations:** ^1^ Murdoch Children's Research Institute, Melbourne, Australia; ^2^Department of Anatomy and Neuroscience, The University of Melbourne, Melbourne, Australia; ^3^Department of Nephrology, Royal Children's Hospital, Melbourne, Australia; ^4^Department of Paediatrics, The University of Melbourne, Melbourne, Australia

## Background

Nephrotic syndrome (NS) is one of the most common monogenic kidney diseases. *NPHS2* missense mutations were reported to result in misfolding and mistrafficking of Podocin. Such studies overexpressed mutant proteins in immortalised podocyte and non-podocyte cell lines, which may not reflect the *in vivo* consequences of the mutation. We generated *NPHS2* mutant iPSC-derived kidney organoids as a model to dissect the pathogenic process of NS.

## Methods

A control human fibroblast line was simultaneously reprogrammed and CRISPR/Cas9 gene edited, generating 5 iPSC lines containing mutations of the endogenous *NPHS2* locus (G92C, P118L, R138Q, R168H, R291W) as well as a control wild-type line. All lines were differentiated into kidney organoids containing all nephron segments. Podocin localisation was assessed using co- immunofluorescence with known markers of subcellular compartments.

## Results

Mutant lines revealed a marked reduction of the Podocin protein compared to control and mutant podocin mislocalisation was often in contradiction with existing literature. G92C and P118L mutant proteins accumulated mainly at the slit diaphragm and the endoplasmic reticulum respectively, as previously described. However, co-staining with GM130 demonstrated a partial Golgi retention for P118L, R168H and R291W. R138Q and R291W were both found to partially accumulate in late endosomes. Finally, all 5 mutants displayed significantly more apoptotic Caspase3+ podocytes than controls.

## Conclusion

Discrepancies between previous reports and our findings highlight the need for a more appropriate model to study the pathobiology of NPHS2 mutations. These organoids will allow us to explore approaches to rescue individual Podocin defects guiding the development of new therapeutic strategies.

# Abstract: 3 First identified GBM modulator (P3H2) causes thin basement membrane nephropathy

**Authors:** Ms. Hande Aypek, Dr. Christoph Krisp, Dr. Oliver Kretz, Prof. Dr. Kerstin Amann, Dr. Kerstin Benz, Prof. Dr. Hartmut Sclüter, Dr. Zheng-mao Hu, Dr. Arif O. Khan, Prof. Dr. Victor G. Puelles, Prof. Dr. Carsten Bergmann, Prof. Dr. Tobias B. Huber, Dr. Florian Grahammer

**Affiliations:** ^1^Department of Medicine, University Medical Center Hamburg-Eppendorf, Hamburg, Germany; ^2^Institute of Clinical Chemistry and Laboratory Medicine, Mass Spectrometric Proteomics Group, University Medical Center Hamburg-Eppendorf, Hamburg, Germany; ^3^Department of Nephropathology, Institute of Pathology, University of Erlangen, Erlangen, Germany; ^4^Department of Pediatrics, University of Erlangen, Erlangen, Erlangen, Germany; ^5^Center for Medical Genetics, School of Life Sciences, Changsha, Hunan, China; ^6^Eye Institute, Cleveland Clinic Abu Dhabi, Abu Dhabi, UAE; ^7^Department of Ophthalmology, Cleveland Clinic Lerner College of Medicine of Case Western University, Cleveland, USA; ^8^Medizinische Genetik Mainz, Limbach Genetics, Mainz, Germany; ^9^Department of Medicine, Division of Nephrology, University Hospital Freiburg, Freiburg, Germany

The Glomerular filtration barrier (GFB) is the decisive unit to generate primary urine. It is composed of a fenestrated endothelium, glomerular basement membrane (GBM), podocytes and slit diaphragm. Collagen IV, laminin-521, nidogen, and agrin are major structural proteins generating the meshwork structure of the GBM. As a scaffold, it supports endothelial cells and podocytes. It promotes the dynamic structure of the GFB in homeostasis. Disruption of the GFB leads to albuminuria and microhematuria.

A *LEPREL1* gene mutation was identified in an albuminuric patient cohort by using an expression-based candidate gene approach. *LEPREL1* encodes Prolyl 3-Hydroxylase 2 (P3H2) a post-translational collagen IV modifier. P3H2 hydroxylases the 3' of prolines in collagen IV in the endoplasmic reticulum. Its relevance for the GFB has not been studied yet. Therefore, we aimed to investigate the role of P3H2 on GFB homeostasis. We generated *Leprel1* podocyte-specific knock-out (KO) mice and a *LEPREL1* KO immortalized human podocyte cell line. Characterization of these mice revealed that the absence of P3H2 induces a thin basement membrane nephropathy (TBMN) phenotype with thinner GBM than wild- type mice and development of focal glomerulosclerosis, podocyte loss and PEC activation over time. Patients carrying *LEPREL1* mutations present with microalbuminuria and microhematuria. Moreover, GBM proteomics of KO mice and ECM proteomics of KO cells indicated a disrupted regulation of collagen IV sub-chains.

In conclusion, P3H2 is the first identified GBM modulator causing a TBMN phenotype. P3H2 affects collagen IV network assembly and stability explaining this phenotype on a molecular level.

# Abstract: 4 DGAT2 inhibition in kidney organoids and podocytes to reduce cytotoxicity of APOL1 kidney risk variants

**Authors:** Dr. Justin Chun, Dr. Cristian Riella, Dr. Hyunjae Chung, Dr. Jia-Yue Zhang, Dr. Minxian Wang, Mr. Jose Magraner, Dr. Guilherme Ribas, Dr. Henrique Ribas, Dr. Seth Alper, Dr. David Friedman, Dr. Martin Pollak

**Affiliations:** ^1^University of Calgary, Calgary, Canada; ^2^Beth Israel Deaconess Medical Center, Boston, USA; ^3^Harvard Medical School, Boston, USA; ^4^BROAD Institute of Harvard and Massachusetts Institute of Technology, Boston, USA; ^5^Federal University of Paraná, Curitiba, Brazil

## Background

Two APOL1 coding variants (G1 and G2) are highly associated with African ancestry and major contributors to the large racial disparity in rates of human kidney disease. We previously demonstrated that recruitment of APOL1 risk variants G1 and G2 to lipid droplets (LDs) leads to reduced APOL1-mediated cytotoxicity in human podocytes.

## Methods

Bulk RNA sequencing was performed for isogenic kidney organoids of APOL1 genotypes G0 and G2. LD formation was altered by inhibiting DGAT1 (diacylglycerol O-acyltransferase-1) and DGAT2 in kidney organoids, human primary podocytes transfected with APOL1 and HEK 293 cells stably expressing APOL1.

## Results

APOL1 is highly upregulated in response to interferon gamma (IFN−y) in kidney organoids of APOL1G2/G2 compared to APOL1G0/G0. There were fewer LDs in APOL1G2/G2 kidney organoids than in APOL1G0/G0 organoids. Bulk RNA sequencing revealed downregulation of genes involved in lipid biosynthesis in APOL1G2/G2 kidney organoids. Interestingly, whereas DGAT1 (diacylglycerol O- acyltransferase-1) inhibition reduced kidney organoid LD number, DGAT2 inhibition unexpectedly increased organoid LD number. Consistent with these findings in organoids, DGAT1 inhibition in both primary podocytes and HEK293 cells stably expressing APOL1 diminished LD formation, while DGAT2 inhibition increased LD formation and promoted recruitment of APOL1 risk variants to LDs to reduced cellular autophagic flux and cytotoxicity.

## Conclusion

Our results show the importance of alterations in LD synthesis and formation as modulators of APOL1-associated cytotoxicity and suggest inhibition of DGAT2 as a potential therapy to attenuate cytotoxic effects of APOL1 risk variants.

# Abstract: 5 Recessive mutations in *SEMA3G* as a potential novel cause of nephrotic syndrome

**Authors:** Dr Florian Buerger, Dr Verena Klambt, Dr Camille Nicolas Frank, Dr Ana Onuchic-Whitford, Dr Ronen Schneider, Dr Konstantin Deutsch, Dr Amar J Majmundar, MSc Shirlee Shril, Dr Friedhelm Hildebrandt

**Affiliations:** ^1^Department of Pediatrics, Boston Children's Hospital, Harvard Medical School, Boston, USA; ^2^Renal Division, Brigham and Women's Hospital, Harvard Medical School, Boston, USA

## Introduction

Nephrotic syndrome (NS) is the second leading cause of chronic kidney disease in the first three decades of life. The identification of monogenic causes of NS has revealed ˜60 single-gene etiologies. While in up to 30% of patients with NS a causative mutation may be detected, many cases remain without molecular diagnosis (Sadowski *JASN* 26:1279, 2015). NS genes are predominantly expressed in glomerular podocytes and the encoded proteins merge onto molecular complexes and pathways that are essential to podocyte development or homeostasis.

## Methods

To identify novel monogenic causes of NS, we performed whole exome sequencing (WES) in an international cohort of 1382 NS patients.

## Results

We identified homozygous mutations in *SEMA3G* in 3 unrelated children with nephrotic syndrome, a nonsense mutation (c.1078C>T, p.Arg360*), an essential splice site mutation (c.460-2A>G, predicted to skipping of exon 5 and thus causing truncation of the protein) and a missense mutation (c.2225G>A, p.Arg742Gln). Interrogating kidney single-cell RNA sequencing datasets, we detected predominant expression of *SEMA3G* in podocytes (Karaiskos *JASN* 29:2060, 2018). When overexpressing all *SEMA3G* variants in cultured podocytes, we observed comparable cellular expression of all variants, secretion and furin-dependent cleavage of wildtype and p.Arg742Gln SEMA3G but no secretion of the two truncating patient variants. We then performed co-immunofluorescence staining in adult rat kidney sections for Sema3g and established markers of glomerular structures. Herein, Sema3g signal was strongest in podocyte foot processes and glomerular basement membranes.

## Conclusions

We, here, identified recessive mutations in *SEMA3G* as a potential novel cause of nephrotic syndrome in children.

# Abstract: 1 Urolithin A is a potent regulator of podocyte functions

**Authors:** Milena Kotewicz, Barbara Lewko, Agnieszka Daca, Professor Mirosława Krauze-Baranowska, Professor Leszek Kalinowski

**Affiliations:** Medical University of Gdansk, Gdansk, Poland

Urolithins are polyphenolic gut microbiota- derived metabolites of dietary elagitannins. Absorbed compounds circulate in plasma and are found in urine. Urolithin A (UA) is the predominant isoform in humans and its biological properties have been demonstrated in the *in vivo* and in the *in vitro* models. Nevertheless, few studies show their effects in the kidney. The aim of our study was to examine if UA influences nephrin expression and the effects of TGFb in podocytes cultured in conditions mimicking diabetes.

Immortalized mouse podocytes were cultured for 7 days in DMEM containing normal (5.5 mM, NG) or high (30 mM, HG) glucose. 10 mM UA and/or 5ng/ml TGFb were added for 24 hours. The cell surface nephrin epitopes and TGFb receptor TBRI were analyzed by flow cytometry (FC). NPHS1 and TBRI mRNA were evaluated by RT-PCR.

UA significantly increased surface nephrin in NG (174±4 vs 73±8 for Control, MFI units, P< 0.001) and in HG (164±25 vs 97±10, P<0.02). In HG, co-incubation of UA with TGFb elevated the surface nephrin, as compared to TGFb alone (P=0.002). FC revealed downregulation by UA of TBRI in NG (by 64%) and in HG (by 57%). NPHS1 mRNA decreased by 50% in HG, whereas TBRI mRNA remained at the level similar to NG. Surprisingly, UA reduced NPHS1 mRNA in NG (by50%) and in HG (by 80%), while TBRI mRNA was elevated by 55% in NG and HG.

Our results show that urolithins can be potent regulators of podocyte functions in normal and diabetic conditions.

# Abstract: 2 Molecular and functional characterization of urine derived *APOL1* G2/G2 high- risk genotype podocyte

**Authors:** Ms. Oyindamola Adebayo, Dr. Pepe Ekulu, Dr. Jean-Paul Decuypere, Dr. Linda Bellucci, Dr. Mohammed Elmonem, Dr. Agathe Nkoy, Prof. Dr. Djalila Mekhali, Prof. Benedetta Bussolati, Prof. Dr. Lambertus van den Heuvel, Dr. Fanny Arcolino, Prof. Dr. Elena Levtchenko

**Affiliations:** ^1^Department of Development and Regeneration, Kathiolieke Universiteit Leuven, Leuven, Belgium; ^2^Centre for Molecular and Vascular Biology, Department of Cardiovascular Sciences, Kathiolieke Universiteit Leuven, Leuven, Belgium; ^3^Division of Nephrology, Department of Paediatrics, University Hospital of Kinshasa, Faculty of Medicine, University of Kinshasa, Kinshasa, Congo, the Democratic Republic of the; ^4^Department of Molecular Biotechnology and Health Sciences, University of Turin, Turin, Italy; ^5^Department of Clinical and Chemical Pathology, Faculty of Medicine, Cairo University, Cairo, Egypt; ^6^Department of Paediatric Nephrology, Radboud University Medical Centre, Nijmegen, Netherlands

## Background

Apolipoprotein L1 *(APOL1)* risk variants, G1 and G2, increase the risk of various non- diabetic kidney diseases in the African population. To date, the precise mechanisms by which *APOL1* risk variants induce injury on podocytes and other kidney cells remain unclear. Trying to unravel these mechanisms, most studies used animal or cell models created by gene editing.

## Methods

Conditionally immortalized human podocyte cell lines from urine of a donor carrying *APOL1* high risk genotype, G2/G2 was developed. The *APOL1* G2/G2 cell lines were characterized for podocyte markers at both the mRNA and the protein levels, using real-time quantitative PCR, Western blot and immunofluorescence staining. Following induction of *APOL1* expression by 50 µg/mL polyinosinic- polycytidylic acid (poly(I:C)), we assessed the functional features of APOL1-induced podocyte dysfunction such as cell detachment, cell viability, cell death, autophagy, cytoskeleton organization and podocyte permeability. As control, *APOL1* wild type (G0/G0) podocytes previously generated from a Caucasian donor were used.

## Results

We successfully generated human *APOL1* G2/G2 urinary podocyte cell lines. Upon exposure to poly(I:C), G2/G2 and G0/G0 podocytes upregulated *APOL1* expression resulting in podocytes detachment, decreased cells viability and increased apoptosis rate in a genotype-independent manner. G2/G2 podocyte cell lines exhibited altered features, including upregulation of CD2AP, alteration of cytoskeleton, reduction of autophagic flux and increased permeability in an *in vitro* model under continuous perfusion.

## Conclusion

The human *APOL1* G2/G2 podocyte cell model is a useful tool for unraveling the mechanisms of APOL1-induced podocyte injury and the cellular functions of APOL1.

# Abstract: 3 Evaluating renal fibrosis in a nephrotoxic nephritis model using DTI-MRI

**Authors:** Dr. Shimrit Avraham, Dr. Luke Xie, DR. Ben Korin, Dr. Mayra Cruz Tleugabulova, Dr. Andrey Shaw Genentech

**Affiliations:** SSF, USA

Kidney function is typically assessed by estimated glomerular filtration rate (eGFR). However, GFR measurements can vary according to the patient background, age, and biological and measurement variability. Magnetic resonance imaging (MRI) offers a method to evaluate the renal structure and function noninvasively in real-time. Here, we used the glomerular immune injury model, nephrotoxic nephritis (NTN), to model chronic kidney disease (CKD) in mice and evaluated disease progression using diffusion tensor MRI (DTI-MRI). NTN was induced by injecting nephrotoxic serum (NTS) and verified by CKD parameters including proteinuria, mesangial expansion, and renal fibrosis. Using DTI-MRI, we were able to detect changes associated with inflammation and interstitial fibrosis. Both the axial diffusivity and the fractional anisotropy decreased over time in accordance with the increased destruction of glomeruli and fibrosis of the kidney found in histology. Our study demonstrates the advantages of DTI- MRI over traditional measurements and can be used for the development of diagnostic techniques for a variety of renal diseases.

# Abstract: 4 Proteomics of mechanically stretched podocytes identifying potential key proteins for glomerular hypertension

**Authors:** Dr. Felix Kliewe, Francescapaola Mattias, Dr. Elke Hammer, Dr. Sabine Ameling, Prof. Dr. Karlhans Endlich, Prof. Dr. Uwe Völker, Prof. Dr. Nicole Endlich

**Affiliations:** ^1^Department of Anatomy and Cell Biology, Greifswald, Germany; ^2^Interfaculty Institute for Genetics and Functional Genomics, Greifswald, Germany

## Background

Glomerular hypertension induces mechanical stress on podocytes, which often leads to podocyte detachment and glomerulosclerosis in patients suffering from hypertension. Although podocytes are known to be mechanosensitive, the key proteins involved are still unknown.

## Methods

Mouse podocytes were cultured on silicone membranes that were connected to the stretch apparatus (NIPOKA GmbH, Greifswald) with 5% and 8% extension for 3 days to simulate physiological and pathological conditions. After mechanical stretch, the cell lysates were measured by LC-MS/MS and interesting candidate proteins were verified by qRT-PCR, Western blot and immunostaining.

## Results

Under physiological stretch conditions, we found 63 significantly down-regulated and 71 significantly up- regulated proteins and under pathological stretch conditions, 269 significantly down- and 193 significantly up-regulated proteins. According to DAVID Functional Annotation, most of the down- regulated proteins are cytoskeleton and actin-binding proteins. In contrast, the up-regulated proteins are more associated in clusters affecting transcription and mRNA processing. To identify rare events such as alternative splicing, we additionally analyzed samples by RNA sequencing.

Moreover, we recultivated podocytes that detached rapidly and the mechanically resistant cells that remained on the stretch membrane. We observed that podocytes showed similar behavior after renewed mechanical stress. Analysis by fluorescence microscopy suggests that this is due to differential expression of focal adhesion proteins.

## Conclusions

Our proteomic data of stretched podocytes *in vitro* identified potential key proteins that may also be involved in the detachment of podocytes *in vivo* as well playing a role in patients suffering from glomerular hypertension.

# Abstract: 5 Development of an improved differentiation protocol for iPSC-derived podocytes

**Authors:** Sergii Kyrychenko, Justin Gallagher, Timothy Norman, Alyssa Fanelli, Xiang Pan, Anna Rachel Gallagher, Thomas Gustafson, Maria D Lalioti Goldfinch Bio

**Affiliations:** Cambridge, USA

## Background

The integrity of podocyte processes ensures a tight slit diaphragm, which, when compromised leads to proteinuria and often renal failure. Despite significant strides in the genetics of renal disease, only a minority of podocytopathies are explained by monogenic mutations, with the rest being attributed to multigenic or environmental factors. Therefore, to develop personalized therapies it would be ideal to use patient-derived cells.

## Methods

Several protocols for the differentiation of iPSCs into podocytes (iPodos) have been published. The biggest problems remain the lack of podocyte maturity and low reproducibility. We compared two published protocols [Ciampi 2016; Musah 2018] and tested additional conditions, including extracellular matrices, media, and length of differentiation.

## Results

Both protocols generated iPodos with similar efficiency, as measured by synaptopodin, nephrin, and podocin staining. iPodos generated from protocol-2 were morphologically homogeneous, developed more filopodia and complex cell-cell junctions, and showed extended survival up to 4 weeks post-differentiation, than those of protocol-1. iPodos from protocol- 1 remained relatively immature, based on the expression of collagen a1a2a1(IV) and lack of a3a4a5(IV).

Response to Protamine sulfate (PS) or Puromycin Aminonucleoside (PAN) treatment was more consistent in protocol 2. PS treatment induced a significant and reproducible dose- and time- dependent decrease in synaptopodin staining, and a robust accumulation of phalloidin aggregation. Both effects were prevented by cyclosporin A, in a similar manner as in mouse podocytes, that were used as a positive control.

# Abstract: Novel Keap-Nrf2 protein-protein interaction inhibitor UBE-1099 ameliorates glomerulosclerosis in Alport syndrome mouse model

**Authors:** Mr. Shota Kaseda, Mr. Yuya Sannomiya, Mr. Jun Kuwazuru, Mary Ann Suico, Ms. Sayaka Ogi, Ms. Ryoko Sasaki, Mr. Hidetoshi Sunamoto, Mr. Hirohiko Fukiya, Mr. Hayato Nishiyama, Misato Kamura, Ms. Saki Niinou, Mr. Jun Horizono, Mr. Yuimi Koyama, Futoshi Nara, Tsuyoshi Shuto, Mr. Kazuhiro Onuma, Prof. Hirofumi Kai

**Affiliations:** ^1^Department of Molecular Medicine, Kumamoto, Japan; ^2^HIGO program, Kumamoto, Japan; ^3^Pharmaceutical Research Laboratory, UBE Industries, Ltd, Yamaguchi, Japan

## Background

Bardoxolone methyl is an electrophilic agent that induces Nrf2 activation by irreversibly and covalently binding to the cysteine residue of Keap1. Bardoxolone methyl has been shown to improve glomerular filtration rate (GFR) in clinical trials and is attracting attention as a novel agent for chronic kidney disease. However, there is concern about long-term efficacy due to the unknown mechanism of GFR improvement and transient increase in albuminuria. Moreover, irreversible Keap1 inhibitors such as Bardoxolone methyl may covalently bind to other proteins in a non-specific manner and induce side effects due to off-target activities.

## Methods

We developed a reversible Keap1 inhibitor that inhibits Keap1-Nrf2 protein-protein interaction (PPI) and evaluated its efficacy using Alport syndrome mice model (Col4a5-G5X). Development of Keap1-Nrf2 PPI inhibitor was performed by fluorescence polarization and Nqo1 induction test. The obtained novel compound UBE-1099 (30 mg/kg/day) and CDDO-Im (3, 10 mg/kg/day; rodent tolerable Bardoxolone methyl analogue) were orally administrated to Alport mice and efficacy was evaluated.

## Results

UBE-1099 showed higher Nqo1 induction efficiency compared with CDDO-Im in mouse renal tissue. While CDDO-Im only improved inflammation pathology in Alport mice, UBE-1099 uniformly improved renal function (GFR and Plasma creatinine, but not albuminuria), podocyte injury, glomerulosclerosis, inflammation and fibrosis. Moreover, UBE-1099 treatment significantly prolonged the lifespan of Alport mice.

## Conclusions

This study firstly revealed the efficacy of Keap1-Nrf2 PPI inhibitor for glomerulosclerosis. We will elucidate next the mechanism of renal pathology improvement, which may provide useful information for Nrf2 activators including bardoxolone methyl for clinical application.

# Abstract: 1 A Precision Medicine Approach to Developing Novel Medicines for Podocytopathies: The GFB-887 Clinical Development Program

**Authors:** Dr. Liron Walsh, Dr. Yossi Dogan, Dr. Youssef MK Farag, Dr. Krishna Padmanabhan, Dr. Leslie Johnson, Mr. John Lawler, Ms. Caitlin Cornwall, Dr. Greggory A. Gaich, Dr. Craig Ostroff, Dr. Debbie S. Gipson, Dr. Hiddo J. L. Heerspink, Dr. Howard Trachtman, Dr. Katherine R. Tuttle, Dr. Frank Czerwiec

**Affiliations:** ^1^Goldfinch Bio, Cambridge, USA; ^2^Cytel, Waltham, USA; ^3^University of Michigan, Ann Arbor, USA; ^4^University of Groningen, Groningen, Netherlands; ^5^NYU Langone Health, New York, USA; ^6^Providence Health Care, Spokane, USA; ^7^University of Washington, Seattle, USA

Our understanding of mechanisms driving podocyte injury increasingly support development of targeted therapies for proteinuric kidney diseases. We describe our approach to developing a targeted therapy for podocytopathies driven by TRPC5-Rac1 pathway overactivation and using urinary Rac1 (uRac1) as a novel predictive and target-engagement biomarker. GFB-887, a podocyte-targeting, subtype selective and potent small molecule TRPC5-inhibitor, was identified as a potential targeted therapy for patients with podocytopathies. In rodent models of focal segmental glomerulosclerosis (FSGS), minimal change disease (MCD) and diabetic nephropathy (DN), GFB-887 reduced both uRac1 and albuminuria. Rac1 was detected in extracellular vesicles isolated from podocyte-conditioned culture media and in human urine, consistent with kidney origin. uRac1 concentrations were increased in patients with FSGS and DN compared to healthy adults indicating a possible predictive biomarker.

In a first-in-human, randomized, double-blind, placebo-controlled, single-ascending dose trial (GFB-887 5 to 900mg), GFB-887 was found to be safe and well tolerated, with a once daily harmacokinetic profile. Statistically significant area under the curve (AUC) reductions in uRac1concentrations were observed from baseline with increasing doses of GFB-887, indicative of TRPC5-Rac1 pathway engagement and inhibition. To efficiently explore the safety and efficacy of GFB-887, and utility of uRac1 as a predictive and target-engagement biomarker, we utilize a basket trial and adaptive study design approach for our phase 2 program. We are currently enrolling adult patients with FSGS, treatment resistance-MCD, and DN in TRACTION-2: a phase 2, randomized, double-blind, placebo-controlled, multiple-ascending dose study of GFB-887 for 12 weeks (NCT04387448).

# Abstract: 2 Developing a small-molecule therapeutic to treat diabetic kidney disease

**Authors:** Javier Varona Santos, Tonrnberg Toni, Travis Lavoi, Marcello Giulianotti, Sandra Merscher, Alessia Fornoni, Hassan Al-Ali

**Affiliations:** ^1^Peggy and Harold Katz Family Drug Discovery Center, University of Miami, Miller School of Medicine, Miami, USA; ^2^University of Miami, Miami, USA; ^3^Torrey Pines Institute for Molecular Studies, Port St. Lucie, USA

## Background

Diabetic Kidney Disease (DKD) is the leading cause of kidney failure worldwide. Injury and loss of podocytes underly the development and progression of DKD. We previously showed that accumulation of lipid droplets (LDs) in podocytes is associated with cell death, and that reducing LD accumulation combats DKD. We hypothesize that a targeted small molecule that directly blocks LD accumulation in podocytes can halt or reverse the progression of DKD.

## Methods

A robust phenotypic assay with human podocytes to identify compounds that reduce LD accumulation in these cells was developed. We screened a chemical library from the Torrey Pines Institute for Molecular Studies (TPIMS) containing >45 million molecules. Structure-activity-relationship (SAR) studies from identified hits were performed. RNAseq libraries from podocytes treated with one of the identified hits in the presence of sera from subjects with or without DKD were generated.

## Results

The assay identified several hits from the TPIMS library. SAR studies showed strong feasibility for hit-to- lead optimization of one series. A representative compound reduced the expression of genes related to TNF-signaling, a pathway associated with LD accumulation and podocyte injury, and the expression of lysosome-associated membrane glycoprotein (LAMP), which correlates with improved autophagy/lipophagy. This compound is currently being tested in a mouse model of DKD.

## Conclusions

Our screen identified a promising molecular series that strongly reverses features of podocyte toxicity and death in DKD. These compounds can now help with the investigation of DKD pathology, identification of novel drug targets, and development of a therapeutic candidate.

# Abstract: Whole exome sequencing reveals a monogenic cause of disease in 26% of 335 families with steroid-resistant nephrotic syndrome

**Authors:** Dr. Konstantin Deutsch, Dr. Ronen Schneider, Dr. Kirollos Yousef, Dr. Florian Buerger, Dr. Camille Nicolas Frank, Dr. Verena Klambt, Dr. Ana C. Onuchic-Whitford, Shirlee Shril, Dr. Shrikant Mane, Dr. Michael J. G. Somers, Dr. Friedhelm Hildebrandt

**Affiliations:** ^1^Boston Children's Hospital, Boston, USA; ^2^Yale University School of Medicine, New Haven, USA

## Background

Steroid-resistant nephrotic syndrome (SRNS) is a common cause of chronic kidney disease *(Nat Rev Dis Primers* 6:68, 2020). 60 monogenic causes of SRNS have been identified *(Nephrol Dial Transplant* 31:1802, 2016). Previously, causative mutations were detected in 25% of patients with SRNS using whole-exome sequencing (WES) *(Clin J Am Soc Nephrol* 13:53, 2018). Recently, WES has become more accessible, with the advantage of detecting not only known causes of SRNS but also novel candidate genes.

## Methods

To reveal the frequency of causative mutations and to identify potential novel disease genes, we evaluated WES data from 335 families with the onset of SRNS under the age of 25 years for causative variants.

## Results

In 87/335 families (26%), we identified a likely causative variant in a known SRNS gene. These variants were found in 22 of the 60 known genes we evaluated. In 58 families (17.3%), we identified mutations in a potential novel candidate gene. In families in whom homozygosity by descent was >60Mbp, we identified causative variants in known SRNS genes in 48.1%, as opposed to 15.4% in individuals with a homozygosity of <60Mbp.

## Conclusion

In this study, we established a molecular genetic diagnosis in 26% of 335 families with SRNS. These results are consistent with earlier findings (J *Am Soc Nephrol* 26:1279, 2015; *Clin J Am Soc Nephrol* 13:53, 2018) and underline the importance of WES in diagnostics for patients with SRNS. We also identified potential novel candidate genes for SRNS.

# Abstract: 1 DAAM2 variants cause nephrotic syndrome via actin dysregulation

**Authors:** Dr. Ronen Schneider, Dr. Konstantin Deutsch, Dr. Gregory Hoeprich, Dr. Jonathan Marquez, Dr. Steve Seltzsam, Dr. Florian Buerger, Dr. Amar Majmundar, Ms. Shirlee Shril, Dr. Mustafa Khokha, Dr. Julia Hoefele, Dr. Bruce Goode, Dr. Friedhelm Hildebrandt

**Affiliations:** ^1^Boston Children's Hospital, Boston, USA; ^2^Brandeis University, Waltham, USA; ^3^Yale University, New Haven, USA; ^4^Technical University of Munich, Munich, Germany.

## Background

The discovery of >60 monogenic causes of nephrotic syndrome (NS) has revealed a central role for the actin regulators RhoA/Rac1/Cdc42 and their effectors, including the formin *INF2.*

## Methods and Results

By whole exome sequencing (WES), we here discovered bi-allelic variants in the formin *DAAM2* in four unrelated families with steroid resistant NS. We show that DAAM2 localizes to the cytoplasm in podocytes and in kidney sections. Further, the variants impair DAAM2-dependent actin remodeling processes: Wildtype-DAAM2 cDNA, but not cDNA representing missense variants found in individuals with NS, rescued reduced podocyte migration rate (PMR), and restored reduced filopodia formation in shRNA-induced DAAM2-knockdown podocytes. Filopodia restoration was also induced by the formin activating molecule IMM-01. DAAM2 also co-localizes and co-immunoprecipitates with INF2, which is intriguing since variants in both formins cause NS. Using *in-vitro* bulk and TIRF microscopy assays, we find that *Daam2* variants alter actin assembly activities of the formin. In a *Xenopus daam2−* CRISPR knockout model, we demonstrate actin dysregulation *in-vivo* and glomerular maldevelopment that is rescued by *WT-DAAM2* mRNA.

## Conclusions

We conclude that *DAAM2* variants are a likely cause of monogenic human SRNS due to actin dysregulation in podocytes. Further, we provide evidence that DAAM2-associated SRNS may be amenable to treatment using actin regulating compounds.

# Abstract: 2 Recessive mutations in SYNPO2 as a candidate of monogenic nephrotic syndrome

**Authors:** Dr. Ronen Schneider, Dr. Youying Mao, Dr. Peter van der Ven, Dr. Marvin Assent, Dr. Florian Buerger, Dr. Konstantin Deutsch, Dr. Amar Majmundar, Dr. Nandini Nagarajan Margam, Dr. Roy Duncan, Dr. Jonathan Marquez, Dr. Mustafa Khokha, Dr. Dieter Fürst, Ms. Shirlee Shril, Dr. Friedhelm Hildebrandt

**Affiliations:** ^1^Boston Children's Hospital, Boston, USA; ^2^Shanghai Children's Medical Center, Shanghai, China; ^3^University of Bonn, Bonn, Germany; ^4^Dalhousie University, Halifax, Canada; ^5^Yale University, New Haven, USA

## Introduction

Most of the approximately 60 genes that if mutated cause steroid resistant nephrotic syndrome (SRNS) are highly expressed in the glomerular podocyte, rendering SRNS a “podocytopathy”. Methods: We performed whole exome sequencing (WES) in 1200 NS patients.

## Results

We discovered homozygous truncating and homozygous missense mutations in *SYNPO2* (synaptopodin-2) (p.Lys1124* and p.Ala1134Thr) in 2 patients with childhood onset nephrotic syndrome (NS). We found SYNPO2 expression in both podocytes and mesangial cells, but notably, immunofluorescence staining of adult human and rat kidney cryosections, indicated that SYNPO2 is mainly localized in mesangial cells. Subcellular localization studies reveal that in these cells SYNPO2 partially co-localizes with a-actinin and filamin A containing F-actin filaments. Upon transfection in mesangial cells or podocytes, EGFP-SYNPO2 co-localized with a-actinin-4, which gene is mutated in autosomal dominant SRNS in humans. SYNPO2 overexpression increases mesangial cell migration rate (MMR), whereas shRNA knockdown reduces MMR. Decreased MMR was rescued by transfection of wild type mouse *SYNPO2* cDNA but only partially by cDNA representing mutations from the NS patients. The increased mesangial cell migration rate (MMR) by SYNPO2 overexpression was inhibited by ARP complex inhibitor CK666. *SYNPO2* shRNA knockdown in podocytes decreased active Rac1, which was rescued by transfection of wild type *SYNPO2* cDNA but not by cDNA representing any of the 2 mutant variants.

## Conclusion

We show that SYNPO2 dysfunction may lead to Rac1-ARP3 dysregulation in nephrotic syndrome patients. Targeting the Rac1-ARP3 pathway can potentially offer a therapeutic approach to this type of SRNS.

# Abstract: 3 Vitamin D3 determines podocyte differentiation in situ and in vitro.

**Authors:** Dr. Tim Lange, Prof. Stefan Simm, Ms. Marie-Christin Böttcher, Dr. Elke Hammer, Ms. Laura Kuhn, Prof. Lars Kaderali, Ms. Claudia Weber, Prof. Karlhans Endlich, Prof. Uwe Völker, Prof. Nicole Endlich

**Affiliations:** ^1^Institute of Anatomy and Cell Biology, University Medicine Greifswald, Greifswald, Germany; ^2^Institute of Bioinformatics, University Medicine Greifswald, Greifswald, Germany; ^3^Interfaculty Institute for Genetics and Functional Genomics, University Medicine Greifswald, Greifslwad, Germany

Podocyte dedifferentiation with the associated morphological rearrangements is a major cause of chronic kidney disease. Since there is no causal therapy, end-stage renal disease makes renal replacement therapy inevitable. Vitamin D3 (VitD) is a promising candidate to overcome this, but it is controversially discussed. In this study, we used a semi-automated screening model to investigated the influence of VitD on podocyte differentiation in situ and in vitro and to identify downstream target genes.

Cultured glomeruli from transgenic mice expressing CFP nephrin promoter-dependently (GlomAssay) were treated with VitD and its analogue calcipotriol and were analyzed by a semi-automated imaging procedure. Additionally, VitD-, calcipotriol- and VitD receptor (VDR) inhibitor (PS121912) -treated glomeruli were investigated by RNA-Seq to reveal the molecular biological effect of VitD on podocyte differentiation. Furthermore, we treated cultured murine podocytes with VitD and calcipotriol to elucidate the morphological and molecular changes by Immunofluorescence, RT-qPCR and Western blot.

VitD−/ calcipotriol-treated glomeruli showed a significantly higher CFP intensity compared to controls, indicating elevated nephrin levels, which was verified by RT-qPCR and Western blot. We found an up- regulation of VDR in VitD- and calcipotriol-treated glomeruli compared to controls. By RNA-Seq, we identified treatment specific molecular patterns in different signaling pathways. Cultured podocytes showed a significant up-regulation of nephrin, VDR and CYP24A1 due to the treatment of VitD. This is accompanied by an altered podocyte morphology due to reorganization of the actin cytoskeleton.

Our results show that VitD influences podocyte differentiation in situ and in vitro based on specific expression patterns.

# Abstract: 4 Recessive NOS1AP variants impair actin remodeling and cause glomerulopathy in humans and mice.

**Authors:** Dr. Amar Majmundar, Dr. Florian Buerger, Dr. Verena Klämbt, Dr. Ronen Schneider, Dr. Konstantin Deutsch, Dr. Neveen Soliman, Dr. Christoph Aufricht, Ms. Shirlee Shril, Dr. Friedhelm Hildebrandt

**Affiliations:** ^1^Department of Medicine, Boston Children's Hospital, Harvard Medical School, Boston, USA; ^2^Department of Pediatrics, Center for Pediatric Nephrology & Transplantation, Kasr Al Ainy Medical School, Cairo University, Cairo, Egypt; ^3^Department of Pediatrics, Medical University of Vienna, Vienna, Austria

## Introduction

Nephrotic syndrome (NS) is the second leading cause of chronic kidney disease in the first three decades of life. Monogenic forms of NS have revealed defined patho-mechanisms *(NDT* 31:1802, 2016) such as dysregulation of the small GTPase CDC42 (Ashraf *Nat Commun* 2018).

## Methods

Whole exome sequencing (WES) was performed in ˜300 SRNS subjects to discover a novel genetic cause. In a human podocyte cell line, we employed active-CDC42 ELISA, live cell imaging, shRNA- mediated downregulation of *NOS1AP,* and cDNA over-expression of *NOS1AP* and CDC42 pathway factors. Nos1ap^Ex3−/Ex3−^ mice were analyzed for urine albuminuria and histologic features of NS.

## Results

We identified homozygous recessive mutations in 2 unrelated children with early onset NS in *NOS1AP* (c.428G>A; p.C143Y and c.345-3T>G; p.I116Afs*4). *NOS1AP* encodes an adaptor protein that interacts with nitric oxide synthase (NOS) and with NOS effectors through its phospho-tyrosine binding domain (PTB) *(Neuron* 28:183, 2000). Overexpression of wildtype (WT) NOS1AP, but not cDNA constructs bearing patient variants, increased active CDC42 and promoted filopodia and podosome formation. Pharmacologic inhibition of CDC42 or its effectors, formin proteins, reduced NOS1AP- induced filopodia formation. NOS1AP knockdown reduced podocyte migration rate (PMR), which was rescued by overexpression of WT Nos1ap but not by constructs bearing patient variants. PMR in NOS1AP knockdown podocytes was also rescued by constitutively active *CDC42^Q61L^* or the formin *DIAPH3.* Nos1ap^Ex3−/Ex3−^ mice recapitulated the human phenotype, exhibiting proteinuria, foot process effacement and glomerulosclerosis.

## Conclusions

These findings demonstrate that recessive NOS1AP variants impair CDC42/DIAPH- dependent actin remodeling and lead to a glomerulopathy in humans and mice.

# Abstract: 5 GWAS and Independently Associated *COL4AA/4A3* Variants with Haematuria in UK Biobank Participants

**Authors:** Dr. Sarah Gagliano, Dr. Andrew Paterson, Dr. Moumita Barua

**Affiliations:** ^1^Faculté de Médecine, Université de Montréal, Montréal, Canada; ^2^Montréal Heart Institute, Montréal, Montréal, Canada; ^3^Division of Epidemiology and Biostatistics, Dalla Lana School of Public Health, Toronto, Canada; ^4^Genetics and Genome Biology, Research Institute at Hospital for Sick Children, Toronto, Canada; ^5^Division of Nephrology, University Health Network, Toronto, Canada; ^6^Department of Medicine, University of Toronto, Toronto, Canada; ^7^Toronto General Hospital Research Institute, Toronto, Canada; ^8^Institute of Medical Sciences, University of Toronto, Toronto, Canada

## Background

*COL4A4* p.Ser969X (rs35138315) is significantly associated with haematuria in the UK Biobank. Additional variants in a 2.2 Mb interval encompassing *COL4A4/4A3* are reported in albuminuria. Whether there is only one causal variant that is in linkage disequilibrium (LD) with all reported signals or multiple independent variants is unclear. Methods: We performed genome-wide and conditional regional analysis to identify variants associated with haematuria in the UK Biobank. To identify independent *COL4A4/4A3* variants, conditional regional analysis was performed in sequential rounds, incorporating previous signals as covariates. If regional variants are in LD, statistical significance for association will be lost. We used both Haplotype Reference Consortium (HRC) imputed genome-wide data and regional TOPMed-imputed data.

## Results

16409 haematuria cases and 379936 controls from the white British subset were used. Variants at 5 other loci *(PDPN, HLA-B, SORL1, PLLP, TGFB1)* met genome-wide significance (p<5E-8). At the *COL4A4/4A3* region, rs35138315 and two additional variants were independently associated with hematuria. These were *COL4A3* Gly695Arg and a common *COL4A4* intron 25 variant (minor allele frequency=0.21). *COL4A3* Gly695Arg was

reported in a GWAS of haematuria using deCODE data (p value=8.27×10^−8^) and has been implicated in Alport syndrome.

## Conclusions

We identify genome-wide loci (n=6, p<5E-8) including independently associated genetic variants in the *COL4A4/4A3* region associated with haematuria. In turn, our analysis reveals potentially new mechanisms and identifies the commonest *COL4A4/4A3* variants in the white British population associated with haematuria.

# Abstract: 6 SRNS-associated *MYO1E* mutations have differential effects on myosin 1e localization, dynamics, and activity

**Authors:** Ms. Pei-Ju Liu, Dr. Laura Gunther, Dr. Diana Perez, Dr. Jing Bi Karchin, Mr. Chris Pellenz, Ms. Sharon Chase, Ms. Maria Presti, Mr. Eric Plante, Dr. Claire Martin, Dr. Svjetlana Lovric, Dr. Christopher Yengo, Dr. Friedhelm Hildebrandt, Dr. Mira Krendel

**Affiliations:** ^1^Department of Cell and Developmental Biology, State University of New York Upstate Medical University, Syracuse, NY 13210, USA; ^2^Department of Cellular and Molecular Physiology, Pennsylvania State University, College of Medicine, Hershey, PA 17033, USA; ^3^Department of Molecular and Cellular Biology, University of Guelph, Guelph, ON, Canada; ^4^Lunenfeld-Tanenbaum Research Institute, Mount Sinai Hospital, Sinai Health System, Toronto, ON, Canada; ^5^Divison of Nephrology, Department of Medicine, Boston Children's Hospital, Harvard Medical School, Boston, MA 02115, USA

## Background

Myo1e is a non-muscle motor protein enriched in the podocyte foot processes. Mutations in *MYO1E* are associated with steroid-resistant nephrotic syndrome (SRNS). Here, we set out to differentiate between the pathogenic and neutral *MYO1E* variants identified in SRNS patients by exome sequencing.

## Methods

Based on protein sequence conservation and structural predictions, two mutations in the motor domain, T119I and D388H, were selected for this study. EGFP-tagged Myo1e constructs were delivered into the Myo1e-KO podocytes via adenoviral infection to analyze Myo1e protein stability, Myo1e localization, and clathrin-dependent endocytosis, which is known to involve Myo1e activity. Furthermore, truncated Myo1e construct was expressed and purified using the baculoviral expression system and used to measure Myo1e ATPase and motor activity *in vitro*.

## Results

Both mutants were expressed as full-length proteins in the Myo1e-KO podocytes. However, unlike wild- type (WT) Myo1e, T119I was not enriched at the junctions or clathrin-coated vesicles (CCVs) in podocytes. In contrast, D388H localization was similar to the WT. Surprisingly, the dissociation of the D388H from cell-cell junctions and CCVs was decreased, suggesting that this mutation affects Myo1e activity. The ATPase activity and the ability to translocate actin filaments were drastically reduced for the D388H mutant, supporting the findings from cell-based experiments.

## Conclusions

The experimental pipeline developed in this study allowed us to determine that the T119I and D388H mutations appear to be pathogenic, and this workflow can be applied to the future characterization of novel *MYO1E* variants associated with SRNS.

# Abstract: 1 High-dimensional characterization of immune cells in the aged kidney

**Authors:** Dr. Ben Korin, Dr. Shimrit Avraham, Dr. Mayra Cruz Tleugabulova, Dr. Andrey Shaw

**Affiliations:** Genentech, South San Francisco, USA

The kidney is a highly dynamic and vibrant environment, populated with various types of immune and non-immune cells that continuously support its structure and function. Kidney function declines with age, yet little is known about the underlying immune processes that may drive kidney dysfunction during normal aging. Here, we utilized high-dimensional analysis methods to identify and characterize immune populations in the aged mouse kidney. In addition to confirming the presence and phenotype of previously described immune populations, we were able to identify significant alterations in specific cell subsets within the kidney. Our study provides an approach for a system-wide view of immune populations in the kidney and may serve as a valuable resource for understanding immune mechanisms that drive regenerative and detrimental processes in the kidney during normal aging.

# Abstract: 2 Immune checkpoint molecule BTLA attenuates inflammation and glomerular damage in experimental glomerulonephritis

**Authors:** Mr Paul Diefenhardt, Mrs Marie Brand, Mr Thomas Schoemig, Mr Bastian Trinsch, Mr Bernhard Schermer, Mr Paul Brinkkoetter, Mr Thomas Benzing, Mr Sebastian Braehler

**Affiliations:** ^1^University Hospital Cologne, Cologne, Germany; ^2^Center for Molecular Medicine Cologne, Cologne, Germany; ^3^Cologne Excellence Cluster on Cellular Stress Responses in Aging Associated Diseases, Cologne, Germany

## Background

Current treatment of the glomerulonephritis (GN) consists of unspecific, highly toxic immunosuppressive therapies with detrimental adverse effects. More specific therapies are therefore warranted. T lymphocytes are not only key players in the nephrotoxic nephritis (NTN) model in rodents, but also during GN in humans, representing potential targets for tailored therapies. As an immune checkpoint molecule, B and T lymphocyte attenuator (BTLA) is crucial in the regulation of T Lymphocyte activation and has been shown to mediate anti-inflammatory effects in other T cell-mediated disease models.

## Methods

NTN was induced in BTLA knock out mice (BTLA-KO) and littermate controls. Regular urine analysis (ACR) was performed throughout the course of the disease. 14 days after NTN induction, blood, kidneys and spleens were harvested for further analysis. Histological assessment of the kidneys was used to evaluate the severity of NTN. Local immune response in the kidney and systemic immunity was analyzed via flow cytometry and qPCR.

## Results

Wild type mice (WT) showed an increased BTLA expression on renal T cells and dendritic cells throughout the course of NTN. No immuno-phenotype was observed in unstimulated BTLA-KO and WT mice. However, BTLA-KO resulted in aggravation of NTN compared to WT. Quantification and characterization of renal immune cells revealed an increase in proinflammatory cells. Interestingly, especially T Lymphocytes were significantly expanded in BTLA-KO mice.

## Conclusion

BTLA attenuates inflammation in experimental GN through suppression of proinflammatory T Lymphocytes. These results build the foundation of a checkpoint inhibitor based therapy of inflammatory glomerular disease.

# Abstract: 3 Targeting the basement membrane in membranous nephropathy

**Authors:** Mrs Maryline Fresquet, Dr Patrick Hamilton, Miss Emily Williams, Dr Stacey Warwood, Professor Paul Brenchley, Professor Rachel Lennon

**Affiliations:** ^1^Wellcome Centre for Cell-Matrix Research, the University of Manchester, Manchester, United Kingdom; ^2^Central Manchester University Hospitals NHS Foundation Trust, Manchester, United Kingdom

Membranous nephropathy (MN) is characterised by immune deposits scattered within a thickened glomerular basement membrane (GBM). It is associated with massive proteinuria and consequent nephrotic syndrome. Current understanding of the human pathology driven by PLA2R antibody accumulation in the GBM is lacking but is likely to be key to disease features associated with MN.

The aim of this study is to define the matrix compositional changes in MN glomeruli using human biopsies.

Glomerular tissue sections from 5 MN patient biopsies and 5 age-matched control samples (healthy tissue from nephrectomy) were collected. ˜75 glomeruli/patient were isolated by laser-microdissection. Proteins from these dissections were prepared for mass spectrometry (MS) using the S-trap trypsin digestion method.

The proteomic dataset analysis identified ˜1600 proteins (129 proteins exclusively found in MN group and 131 in the control group). PLA2R1, IGHG4 and complement factors were all significantly upregulated in MN group. A subset of 161 matrix proteins were detected. Initial findings suggest an increase in collagens, and the basement membrane laminins. ECM glycoproteins such fibronectin, nephronectin, tenascin-C and vitronectin were all upregulated in the MN samples. In contrast, the ECM regulators cystatin-S and cathepsins were significantly down-regulated. Similar matrix changes were observed in our *in vitro* MN cell-culture model. These differences will be further characterised using tissue-based imaging mass cytometry.

Analysis of the matrix composition (MN glomeruli and cell-derived) using MS revealed key proteins potentially involved in the GBM dysregulation. These results will identify new molecular cues for the onset of pathology associated with MN.

# Abstract: Impact of tryptophan catabolism dysregulation on podocytes in vitro, as well as on the glomerular filtration barrier in zebrafish

**Authors:** Dr. Patricia Bolanos-Palmieri, Dr. Heiko Schenk, Dr. Ahmed Kotb, Dr. Patricia Schroder, Dr. Mario Schiffer

**Affiliations:** ^1^Uniklinikum Erlangen, Erlangen, Germany; ^2^Hannover Medical School, Hannover, Germany; ^3^MDI Biological Laboratory, Maine, USA

## Background

The kynurenine pathway catabolizes tryptophan resulting in the synthesis of NAD and other bioactive metabolites. These metabolites are involved in diverse cellular processes and changes in their abundance are linked to the onset and severity of many pathologies. As the kynurenine enzymes are expressed in the kidney and the metabolites are cleared via the urine, we aim to describe the effects of changes in tryptophan catabolism on glomerular cells.

## Methods

Dysregulation of tryptophan catabolism by morpholino knockdown or inhibition of the enzyme Kmo was done in a transgenic zebrafish line with a circulating GFP-labelled reporter protein. Edema severity and GFP signal were recorded at 96 hours post-fertilization. In this model, a reduction in fluorescence is taken as an indicator of proteinuria. As KMO is located in the mitochondria and the pathway participates in NAD synthesis we also assessed podocyte energy metabolism following KMO inhibition.

## Results

Knockdown or inhibition of the enzymes in the pathway leads to an increase in upstream kynurenine metabolites. Changes in tryptophan catabolism result in edema, pericardial effusion, Bowman's space enlargement, alterations in podocyte markers, and proteinuria in the treated larvae. Moreover, KMO inhibition in podocytes leads to a reduction in cell size and loss of focal contacts, REDOX imbalances, and alterations in mitochondrial function.

## Conclusion

Our results suggest that a dysregulation of the kynurenine pathway may lead to alterations in podocyte structure as well as a shift in metabolic flexibility which may have a negative impact on glomerular filtration barrier integrity.

# Abstract: 1 Altered Rab-Rabphilin system in injured human podocytes stressed by glucose overload and angiotensin II

**Authors:** Olga Martinez-Arroyo, Ana Ortega, Belen Sanchez-Garcia, Ana Flores-Chova, Miriam Lluesma, Josep Redon, Raquel Cortes

**Affiliations:** ^1^Cardiometabolic and Renal Risk Research Group, Biomedical Research Institute Clinic Hopsital - INCLIVA, Valencia, Spain; ^2^Internal Medicine, Valencia, Spain

## Background

Similarities between podocytes and neurons have been described, both are differentiated cells, have a common cytoskeletal organization and machinery of process formation. Therefore, Rab GTPases could have an important role in the regulation of vesicular transport in podocytes. The aim of the present study was to analyze the Rab-Rabphilin system in human podocytes and the impact of high glucose and angiotensin II. Furthermore, alterations in urine cell pellets from hypertensive and diabetic patients were also analyzed. Methods: Apoptosis was analyzed in podocytes, and mRNA level quantification, Western blot analysis, and immunofluorescence were developed to quantify podocyte-specific molecules and Rab-Rabphilin components (Rab3A, Rab27A, and Rabphilin3A). Quantitative RT-qPCR was performed on urinary cell pellet from patients.

## Results

Differentiated podocytes had reduced protein levels of the Rab-rabphillin system compared with undifferentiated cells. After glucose overload and angiotensin II treatment, apoptosis was increased and podocyte-specific proteins were reduced. Rab3A and Rab27A protein levels were increased under glucose overload, and Rabphilin3A decreased. Furthermore, this system exhibited higher levels under stress conditions in a manner of angiotensin II dose and time treatment. Immunofluorescence imaging indicated different expression patterns of podocyte markers and Rab27A under treatments. Finally, Rab3A and Rab27A were increased in patient urine pellets and showed a direct relationship with albuminuria.

## Conclusions

Collectively, these results suggest that the Rab-Rabphilin system could be involved in the alterations observed in injured podocytes under hypertension and diabetes conditions, and that a mechanism may be activated to reduce damage through the vesicular transport enhancement directed by this system.

# Abstract: 2 Crumbs2: a novel key component of the slit diaphragm

**Authors:** Annika Möller-Kerutt, Juan E. Rodriguez-Gatica, Karin Wacker, Rohan Bhatia, Dr. Jan-Peter Siebrasse, Nanda Boon, Dr. Veerle Van Marck, Prof. Dr. Peter Boor, Prof. Dr. Ulrich Kubitscheck, Prof. Dr. Jan Wijnholds, Prof. Dr. Hermann Pavenstädt, Prof. Dr. Thomas Weide

**Affiliations:** ^1^Internal Medicine D, Department of Molecular Nephrology, University Hospital of Muenster, Münster, Germany; ^2^Institute of Physical and Theoretical Chemistry, Department of Biophysical Chemistry, Rheinische Friedrich Wilhelms University Bonn, Bonn, Germany; ^3^Leiden University Medical Center, Department of Ophthalmology, Leiden, Netherlands; ^4^Gerhard-Domagk Institute of Pathology, University Hospital of Muenster, Münster, Germany; ^5^nstitute of Pathology, Department of Nephrology and Immunology, RWTH Aachen University Hospital, Aachen, Germany; ^6^Leiden University Medical Center, Department of Ophthalmology, Leiden, Germany

## Background

Similar to Nephrin, the transmembrane protein Crumbs2 (Crb2) shows prominent expression in glomerular podocytes. Recently, *CRB2* missense mutations were identified as causative of steroid- resistant nephrotic syndrome (SRNS). However, the role of Crb2 at the glomerular filtration barrier (GFB) and its disease mechanisms in patients are still unknown.

## Methods

We generated a podocyte specific knockout mice model (Crb2^podKO^) to study Crb2's role for the GFB. The mice phenotype was analysed on transcriptome level, histologic as well as transmission and scanning electron microscopy. Furthermore, high-resolution expansion microscopy was used to investigate Crb2 and Nephrin distribution in murine glomeruli. Stable cell lines, inducible expressing CRB2 wild type and mutants, were established for interaction studies, live cell imaging, glycosylation and transcriptome analyses.

## Results

Crb2^podKO^ mice showed proteinuria from birth later followed by prominent foot process effacement and glomerulosclerosis combined with upregulation of renal injury and inflammatory markers. Expansion microscopy revealed that Nephrin and Crb2 are localized in adjacent clusters. Strikingly, pull-down assays demonstrated an interaction between their extracellular protein domains. *In vitro,* CRB2 wild type showed enrichment at the cell surface and in cell-cell junctions. In contrast, SRNS-associated CRB2 protein variants and an O-glycosylation defective mutant accumulated in the ER, accompanied by an altered glycosylation and ER stress response.

## Conclusion

Our data suggest that loss of Crb2 localization at the GFB and increasing ER stress are critical factors for onset and progression of CRB2-related SRNS. Thus, Crb2 is probably functioning in an interplay with Nephrin as essential element of the slit diaphragm.

# Abstract: 3 TrkC is necessary for glomerular integrity and involved in Igf1R signalling

**Authors:** Dr. Carolin Lepa, Dr. Sascha Hoppe, Prof. Dr. Giuliano Ciarimboli, Dr. Maja T. Lindenmeyer, Prof. Dr. Clemens D. Cohen, Prof. Dr. Peter Boor, Prof. Dr. Thomas Weide, Prof. Dr. Hermann Pavenstädt, Dr. Britta George

**Affiliations:** ^1^Medizinische Klinik und Poliklinik D, University Hospital Münster, Münster, Germany; ^2^III. Medizinische Klinik und Poliklinik, University Hospital Hamburg-Eppendorf, Hamburg, Germany; ^3^Klinik für Nieren-, Hochdruck-und Rheumaerkrankungen, München Klinik Harlaching, München, Germany; ^4^Institut für Pathologie, University Hospital Aachen, Aachen, Germany

## Background

Podocytes play a central role in the pathogenesis of many glomerular diseases. Podocytes share morphological features with neurons. In both cell types, similar cues guide and maintain cellular processes, such as the tropomyosin-related kinase C (TrkC). TrkC is located at the podocyte intercellular junction and conveys signals to the actin cytoskeleton. *In vivo,* its function in the kidney remains to be defined.

## Methods

C57BL/6 TrkC flox or stop flox mice were crossed with C57BL/6 Six2-Cre mice to create a TrkC knockout *(TrkC-KO)* or TrkC overexpression *(TrkC-OE)* in podocytes, parietal epithelial cells, proximal and distal tubular cells. TrkC signaling mechanisms were studied in murine immortalized podocytes.

## Results

*TrkC-KO* and *TrkC-OE* mice showed proteinuria, podocyte foot process effacement, mesangial proliferation, and aspects of focal segmental glomerulosclerosis during aging. Phosphoproteins associated with insulin, erb-b2 receptor tyrosine kinase (Erbb), and Toll-like receptor signaling were enriched in lysates of podocytes treated with the TrkC ligand neurotrophin-3 (Nt-3). Activation of TrkC by Nt-3 induced phosphorylation of the insulin-like growth factor receptor 1 (Igf1R) on activating tyrosine residues in murine podocytes. Igf1R phosphorylation was increased in kidneys of *TrkC-OE* mice, while it was decreased in kidneys of *TrkC-KO* mice. TrkC expression was elevated in glomerular tissue of patients with diabetic nephropathy compared with control glomerular tissue. TrkC-dependent trans- activation of Igf1R was abolished in cultured podocytes expressing a phosphorylation-deficient mutant of TrkC where tyrosine residue 516 was replaced by phenylalanine.

## Conclusion

Our results identify TrkC to preserve glomerular integrity and trans-activate Igf1R signaling

# Abstract: 4 Deep Learning-based Automated Multi-parameter Analysis of Podocyte Substructure Reveals Differential Patterns of Effacement across Diagnoses

**Authors:** David Unnersjö-Jess, Linus Butt, Martin Höhne, German Sergei, Anna Witasp, Annika Wernerson, Jaakko Patrakka, Peter F Hoyer, Bernhard Schermer, Katarzyna Bozek, Thomas Benzing

**Affiliations:** ^1^University Hospital of Cologne, Cologne, Germany; ^2^University of Cologne, Cologne, Germany; ^3^Karolinska Institutet, Stockholm, Sweden; ^4^Karolinska Institutet, Huddinge, Sweden; ^5^University of Duisburg-Essen, Essen, Germany

## Background

Previously, many protocols have been proposed for imaging podocyte substructure (foot processes) using optical microscopy, giving unique opportunities for quantifying podocyte morphology in health and disease. However, all these strategies still involve manual work, impeding throughput and sensitivity.

## Methods

Novel sample preparation protocols were applied to image foot processes in three dimensions using both super-resolution and conventional microscopy in samples prepared using different fixation/preservation protocols. Deep learning-based segmentation was utilized to automatically segment both the slit diaphragm (SD) pattern as well as several thousands of individual foot processes (FPs) per sample.

## Results

Both the SD and FPs were automatically segmented at high accuracy and with higher throughput as compared to previous semi-automatic analyses. Several morphometric parameters were extracted from FPs in a mouse model for FSGS, showing high agreement with our previous analysis strategies. We dubbed this imaging/segmentation approach AMAP (Automatic Morphological Analysis of Podocytes). We performed AMAP on several human patients with different types of kidney disease and, strikingly, found different patterns of effacement for different disease types.

## Conclusions

Three-dimensional optical imaging combined with deep learning segmentation can be used to perform detailed and sensitive morphometric analysis of foot processes with high throughput. This more complete and multi-parametric analysis reveals that the foot process effacement patterns differ between different types of kidney disease. We believe that this might provide an important tool not only as an additional readout in diagnostics, but also to advance our understanding regarding the connection between foot process morphology and albumin filtration.

# Abstract: 5 Renal progenitor-derived extracellular vesicles ameliorate podocyte function in a human in vitro model of glomerular permeability

**Authors:** Lola Buono, Corinne Iampietro, Dr Licia Peruzzi, Prof Silvia Deaglio, Prof Benedetta Bussolati

**Affiliations:** ^1^Molecular Biotechnology Center, Turin, Italy; ^2^A.O. Città della Salute e della Scienza di Torino - Ospedale Regina Margherita, Turin, Italy; ^3^Diparti mento di Scienze Mediche, Turin, Italy

## Background

Idiopathic Nephrotic Syndrome is the most common glomerular disease in children. The large majority of patients are steroid sensitive, while 10-20% of children appear resistant, and frequently progress to end-stage renal disease. Extracellular vesicles (EVs) are cell-released nanosized vesicles involved in cell- to-cell communication, with therapeutic interest for their ability to transfer genetic material and reprogram target cells. We aimed to test the therapeutic potential of renal progenitor-derived EVs on podocytes obtained from children affected by steroid resistant glomerular diseases with characterized genetic mutations.

## Methods

Freshly collected urine derived from nephrotic patients or healthy pregnant women were centrifuged and pelleted, podocytes were cultured and characterized. EVs were isolated from an immortalized renal progenitor cell line derived from a 30-week preterm neonate urine. Serum derived EVs were used as control. Podocytes were seeded on culture inserts and a filtration assay was performed by measuring the transit of FITC-albumin in presence or absence of EVs or of different drugs. Total RNA sequencing was performed on podocytes untreated and treated with EVs.

## Results

Treatment with the renal progenitor-derived EVs was able to significantly reduce permeability in all the steroid-resistant patients. Total RNA sequencing unravelled modulation of several genes involved in stabilization of slit diaphragm specific proteins, common to the different podocyte lines.

## Conclusions

We established a human *in vitro* model that functionally reproduces the podocyte filtration barrier, as a model for personalized medicine. We identified renal progenitor-derived EVs as regulators of podocyte permeability in genetic-driven steroid resistant glomerulonephritis, and the molecular mechanism involved.

# Abstract: 6 Plin5 deficiency in podocyte negatively affects the communication between lipid droplets and mitochondria in Alport Syndrome

**Authors:** Dr. Jin-Ju Kim, Ms. Sydney Wilbon, Dr. Judith Molina David, Dr. Hazel Szeto, Dr. Flavia Fontanesi, Dr. Jeffrey Miner, Dr. Sandra Merscher, Dr. Alessia Fornoni

**Affiliations:** ^1^University of Miami, Miami, USA; ^2^Social Profit Network, Menlo Park, USA; ^3^Washington University School of Medicine, St. Louis, USA

Alport Syndrome (AS) is a hereditary disease caused by collagen type IV mutation. Pathogenic renal lipid droplets (LD) and triglyceride (TG) accumulation have been demonstraded in experimental AS (Col4a3KO mice). Excessive FFA catabolism resulting from excessive lipolysis of TG is a major contributor to cell lipotoxicity. Perilipin5 (PLIN5) regulates TG lipolysis and the interactions between LD and mitochondria, where it protects mitochondria from excessive exposure to FFA. Here we test the hypothesis that PLIN5 deficiency in AS causes excessive TG breakdown and the loss of LD-mitochondrial contact, thus contributing to kidney failure.

Immortalized AS podocytes and WT podocytes were established and characterized in our laboratory by breeding the Col4a3KO mice to H-2kb-tsA58 transgenic mice (Charles River). PLIN5 expression was determined by RT-PCR and WB analysis. TG lipolysis and FFA quantification were determined and normalized to protein content. LD-Mitochondrial contact was determined by TEM analysis.

PLIN5 deficiency was observed in the kidney cortex of Col4a3KO mice (p<0.001) and AS podocytes (p<0.01) compared to controls. AS podocytes showed an increase in TG lipolysis (p<0.05), intracellular FFA (p<0.05) and apoptosis (p<0.01) and reduced in LD-mitochondrial contacts (p<0.05) compared to WT podocytes, a phenotype that was restored after PLIN5 overexpression. Moreover, Ezetimibe, which restored LD-mitochondrial contact *in vitro* (p<0.05) and improved kidney function *in vivo,* was found to restore PLIN5 expression *in vitro* and *in vivo* (p<0.05).

Our study suggests that podocyte PLIN5 deficiency may cause podocyte injury in AS through excessive TG lipolysis and inefficient FA transfer from LD to mitochondria leading to mitochondrial dysfunction and contributing to disease progression.

# Abstract: 1 The influence of diabetic milleau on phosphate homeostasis in podocytes

**Authors:** Tomasz Kulesza, Marlena Typiak, Patrycja Rachubik, Agnieszka Piwkowska

**Affiliations:** ^1^Laboratory of Molecular and Cellular Nephrology, Mossakowski Medical Research Institute Polish Academy of Sciences, Gdansk, Poland; ^2^Faculty of Chemistry, Univeristy of Gdansk, Gdansk, Poland

Phosphate (Pi) homeostasis alteration is commonly present complication in the course of diabetes. Chronic hyperphosphatemia contributes directly to the development of diabetic nephropathy (DN). Pi retention in the body also leads to pathological calcification of soft tissues, which occurs in the final stage of chronic kidney disease caused by DN. Bearing in mind that changes in phosphate balance disturb kidney function, we examined how the diabetic environment affects the Pi equilibrium in podocytes - cells whose proper functioning ensures physiological glomerular filtration and which are the first element damaged during DN.

We established that human immortalized podocytes express mRNA and protein of phosphate transporters - NaPi 2c (SLC34A3), Pit 1 (SLC20A1) and Pit 2 (SLC20A2), which are sodium-dependent and mediate intracellular Pi transport, as well as XPR1, which is responsible for extracellular transport. We found that cells grown in a medium with high glucose concentration (30 mM, HG) expressed less Pit 1 mRNA (real-time PCR) and protein (western blot) than podocytes cultured in a medium with a standard glucose concentration (11 mM, SG). The amount of Pit 2 protein is also decreasing in HG. We determined that the localization of Pit 1 and Pit 2 in cell membrane declined in HG (cell surface biotinylation). We also found that in HG the activity of tissue nonspecific alkaline phosphatase increases, causing a rise in Pi generation.

The above data suggest that hyperglycemic conditions enhance the production of Pi in the podocytes and its retention in the extracellular space, which may induce glomerular calcification.

# Abstract: 2 Spatial Transcriptomic Profiling of Collapsing Glomerulopathy

**Authors:** Dr. Shreeram Akilesh, Dr. Kammi Henriksen, Dr. Roberto Nicosia, Dr. Charles Alpers, Dr. Kelly Smith

**Affiliations:** ^1^University of Washington, Seattle, USA; ^2^University of Chicago, Chicago, USA

## Background

Collapsing glomerulopathy is a histologically distinct variant of focal and segmental glomerulosclerosis that presents with heavy proteinuria and portends a poor prognosis. Collapsing glomerulopathy can be triggered by viral infections such as HIV and SARS-CoV-2. However, it is not known if distinct molecular mechanisms drive histologically indistinguishable lesions of collapsing glomerulopathy in different clinical contexts.

## Methods

Transcriptional profiling of collapsing glomerulopathy lesions is difficult since only a few glomeruli may exhibit this histology within a kidney biopsy. Therefore, we used recently developed spatial transcriptional profiling to quantify 1852 transcripts in individual glomeruli from HIV and SARS- CoV-2 infected patients with biopsy confirmed collapsing glomerulopathy.

## Results

We compared transcriptional signatures on the basis of disease or histology and identified distinct pathways of injury in HIV and SARS-CoV-2 associated collapsing glomerulopathy and thrombotic microangiopathy. Focused validation using immunohistochemistry and RNA *in situ* hybridization showed good concordance with spatial transcriptional profiling results.

## Conclusion

Spatial transcriptional profiling represents a powerful new method to dissect transcriptional programs of pathologically discernible kidney lesions.

# Abstract: 3 Decreased urinary levels of Sirt1 in podocytes as non-invasive biomarker of early renal damage in hypertension

**Authors:** Ana Ortega, Olga Martinez-Arroyo, Ana Flores-Chova, Belen Sanchez-Garcia, Miriam Lluesma, Josep Redon, Raquel Cortes

**Affiliations:** ^1^Cardiometabolic and Renal Risk Research Group, Biomedical Research Institute Clinic Hospital-INCLIVA, Valencia, Spain; ^2^Internal Medicine, Clinic Hospital, Valencia, Spain

## Background

Sirtuin1 (SIRT1) has become an important player in renal damage in hypertension and diabetes, contributing to podocytes injury. The aims of the study were to evaluate the levels of SIRT1 and two miRNAs that regulate SIRT1 expression in podocytes under overload glucose and angiotensin II. In addition, we analyzed their value as biomarkers in hypertensive patients with incipient renal damage with and without diabetes.

## Methods

We quantified urinary SIRT1 and claudin1 mRNA and miR-34a and miR-200a levels by quantitative real-time polymerase chain reaction (RT-qPCR) from patients and in cultured podocytes treated with high glucose and angiotensin II. Western blot and fluorescence analyses were also performed to analyze SiRT1 cell distribution.

## Results

In vitro experiments showed mRNA and protein SIRT1 overall decreases in podocyte cultures under treatment conditions. Immunofluorescence analysis of SIRT1 showed protein accumulations and evidence decreased signal and more granulated cell pattern under both conditions. We also found decreased SIRT1 levels in patients with increased urinary albumin excretion (UAE), the lowest with diabetes presence, and a strong association with UAE, discriminating incipient renal damage. In urine samples, miR-34a was reduced and miR-200a increased, both related to UAE levels. However, both miRNAs were generally increased in podocyte cultures under high glucose and angiotensin-II treatment.

## Conclusions

These results show a significant urinary SIRT1 decrease in albuminuric hypertensive patients, strongly associated with albuminuria. Alterations found in miR-34a and miR-200a both in patients and podocytes cultures reveal novel points to further address their implication in renal injury through SIRT1 regulation.

# Abstract: 4 Oxysterol-binding protein like 7 in chronic kidney disease

**Authors:** Dr. Jeffrey Pressly, Dr. Javier Verona Santos, Dr. Mengyuan Ge, Dr. Sandra Mercher, Dr. Alessia Fornoni

**Affiliations:** University of Miami, Miller School of Medicine, Miami, Florida, USA

## Background

ATP-binding cassette transporter A1 (ABCA1)-mediated podocyte injury plays a role in the progression of glomerular disease and pharmacological inducers of ABCA1 (ABCA1i) are sufficient to rescue glomerular injury in proteinuric mice. These ABCA1i's compete with oxysterol binding to oxysterol-binding protein (OSBP) like 7 (OSBPL7). OSBPs are implicated in cholesterol transfer from the endoplasmic reticulum (ER) to the Golgi, cholesterol efflux and the regulation of ABCA1 expression. However, if OSBPL7 is expressed in the kidney and if it is involved in the preservation of ER function has not been explored. Methods: We utilized podocytes and tissues obtained from wildtype and Col4a3−/− mice, an experimental model of CKD. siOSBPL7 Podocytes and HEK293 cell lines were established using siRNA yielding these cells deficient in OSBPL7. OSBPL7 levels were determined from kidney cortex and podocytes from WT and Col4a3−/− mice by WB, IHC, and RT-PCR. siOSBPL7 podocytes and HEK cells were analyzed for changes in ER stress markers, reactive oxygen species (ROS), cytotoxicity, and apoptosis.

## Results

OSBPL7 is expressed in podocytes. OSBPL7 protein levels are reduced in kidney cortex of Col4a3−/− mice. siOSBPL7 podocytes and HEK293 cells show increased levels of ER stress, ROS, cytotoxicity, and apoptosis. Overexpression of OSBPL7 in Col4a3-/-podocytes lead to a reduction in apoptosis levels further indicating a beneficial role of OSBPL7 in podocytes.

## Conclusion

This study represents the first time that OSBPL7 has been implicated in CKD. OSBPL7 deficiency in podocytes leads to ER stress and ultimately apoptosis suggesting that OSBPL7 levels are beneficial to podocyte function.

# Abstract: 5 Can Podocyte number and density predict the response to therapy in patients with Primary FSGS?

**Authors:** Ms. Natasha De Zoysa, Dr. Jonathan Ling, Dr. Kotaro Haruhara, Professor Peter Kerr, Professor David Nikolic-Paterson, Professor John Bertram, Dr. Luise Cullen-McEwen

**Affiliations:** ^1^Biomedicine Discovery Institute and Department of Anatomy and Developmental Biology, Monash University, Clayton, Australia; ^2^Department of Nephrology, Monash Health, Clayton, Australia; ^3^Division of Nephrology and Hypertension, Jikei University School of Medicine, Tokyo, Japan

## Background

Podocyte loss is critical in primary focal segmental glomerulosclerosis (FSGS). Standard first- line therapy for patients with primary FSGS involves steroid therapy; however steroid resistance occurs in 40-70% of patients. Animal studies have shown that steroid efficacy is achieved partly through preservation of podocyte number. This study aimedto determine if podocyte number and density can predict the response to therapy in patients with primary FSGS.

## Methods

A retrospective cohort study of renal biopsies was conducted from 2009-2020 in Melbourne, Australia at a tertiary hospital (Monash Medical Centre). Patients diagnosed with primary FSGS were screened (n=84). 65 patients were excluded. Included patients were allocated into groups of treatment responders (n=11) or non-responders (n=8) based on data

6 months following initial diagnosis and commencement of treatment. Biopsies were immunofluorescently stained for podocyte-specific markers and used to estimate podometrics. Sections were re-stained with PAS for glomerulosclerotic index (GSI) analysis.

## Results

Podocyte number per glomerulus in responders (347 (215-606); median (IQR)) was 45% higher than in non-responders (190; 143-263) (P=0.03). Podocyte density in responders (76; 58-142 per 106µm3of glomerular volume) was similar to non-responders (66; 44-88 per 106µm3of glomerular volume) (P=0.38). GSI was significantly higher in non-responder patients (1.1; 0.6-2.3) than responders (0.6; 0.2-0.9) (P=0.04), and was significantly and negatively correlated with podocyte number (r = −0.64; P=0.003) and podocyte density (r = −0.48; P=0.04).

## Conclusion

Podocyte number per glomerulus in diagnostic renal biopsies could be used as a predictor of treatment response for patients with primary FSGS.

# Abstract: 6 Using Drosophila melanogaster to study novel FSGS-associated ACTN4 mutations

**Authors:** Johanna Odenthal, Bodo B Beck, Bernhard Schermer, Thomas Benzing, Paul T Brinkkoetter, Malte P Bartram

**Affiliations:** ^1^Department II of Internal Medicine and Center for Molecular Medicine Cologne, University of Cologne, Faculty of Medicine and University Hospital Cologne, Cologne, Germany; ^2^CECAD, University of Cologne, Faculty of Medicine and University Hospital Cologne, Cologne, Germany; ^3^Institute of Human Genetics, University of Cologne, Cologne, Germany; ^4^Center for Molecular Medicine Cologne, University of Cologne, Cologne, Germany

## Background

The actin cross-linking protein Alpha-actinin4 (ACTN4) plays a crucial role in podocyte architecture and function and mutations in the gene are associated with FSGS. Here, performing gene panel sequencing in a pediatric patient presenting with SRNS and FSGS, a *de novo,* potentially disease causing variant of *ACTN4* was identified, which was not found in available genome or exome databases. Our aim is to elucidate the pathogenic potential of this variant for podocytes and FSGS progression.

## Methods

To elucidate pathogenic effects of the identified ACTN4 variant, we employed the *Drosophila* toolbox. We analyzed cell-specific RNAi-mediated knockdown of *Drosophila* Actinin in nephrocytes, podocyte-equivalent cells in the fly, and its impact on cell function and morphology. Rescue experiments with the novel human ACTN4 variant will give indication about possible pathogenic consequences of the mutation when compared to wildtype as well as previously described disease-associated variants of ACTN4.

## Results

Knockdown of *Drosophila* Actinin in nephrocytes leads to severe functional defects, as filtration capacity is diminished by up to 50%. Morphologically, mislocalization of the ZO-1 homologue Polychaetoid was observed as well as overall reduction of nephrocyte diaphragms. First rescue experiments with wildtype human ACTN4 led to partial rescue of functional and morphological phenotypes observed upon Actinin knockdown.

## Conclusion

Our results underline the importance of Actinin for nephrocyte biology. Capacity of wildtype human ACTN4 in rescuing the knock-down associated phenotypes indicates the model's suitability. Further experiments will be performed to elucidate the pathogenicity of the novel ACTN4 variant also in comparison to previously described pathogenic mutations.

# Abstract: 7 Metabolic dysfunctions of podocytes in cystinosis

**Authors:** Mr. Sante Princiero Berlingerio, Prof. Lambertus van den Heuvel, Prof. Marc Fransen, Prof. Dr. Elena Levtchenko

**Affiliations:** ^1^Laboratory of Pediatric Nephrology, KULeuven, Leuven, Belgium; ^2^Laboratory of Peroxisome Biology and Intracellular Communication, KULeuven, Leuven, Belgium

## Background

Cystinosis is a rare, incurable, autosomal recessive disease caused by mutations in the CTNS gene, which encodes the lysosomal cystine transporter cystinosin. In addition to the well- established proximal tubular injury in cystinosis, we showed that cystinosis also affects the glomerulus, and causes podocyte detachment and loss in urine and glomerular proteinuria. Cysteamine decreases cystine accumulation, but it does not reverse proximal tubular and glomerular injury. This suggests that other mechanisms, beyond cystine accumulation, are involved in cystinosis. In this study, we characterized the metabolic and the oxidative status of cystinosis podocytes.

## Methods

Podocytes from cystinosis patients *(CTNS^-/−^* podocytes) and from healthy subjects were used. To evaluate central carbon metabolism, we performed static and labelled metabolomics analysis. To evaluate the redox state, we used gene expression profiling and chemical assays, while the subcellular dynamic redox state was evaluated using roGFP2 probes.

## Results

*CTNS^-/−^* podocytes show peculiar metabolic alterations, with increased levels of intracellular glucose and impaired glycolytic pathway. In addition, they display altered oxidative stress response and impaired mitochondrial function, resulting in decreased abundances of TCA cycle and energy metabolites.

## Conclusions

Cystinosis podocytes show signs of altered energy metabolism and increased oxidative stress. Understanding these mechanisms may pave the way for developing novel therapies in cystinosis.

# Abstract: 8 The Role of Foot Process Orientation in Albumin Filtration

**Authors:** David Unnersjö-Jess, Amer Ramdedovic, Linus Butt, Ingo Plagmann, Bernhard Schermer, Thomas Benzing

**Affiliations:** University Hospital of Cologne, Cologne, Germany

## Background

It has been debated for many decades whether foot processes display a preferred orientation with respect to the orientation of glomerular capillaries. To date, this question has been left unanswered, probably due to limitations of previously available imaging techniques.

## Methods

We applied a recently published imaging protocol, which allows for super-resolved 3D imaging of whole glomerular capillaries. In this way, the angular distribution of several hundreds of primary processes and several thousands of secondary foot processes with respect to the capillary orientation was determined.

## Results

Strikingly, we found a clear preference for primary processes to be aligned perpendicularly to the capillary orientation, whereas foot processes aligned preferably in parallel with the capillary orientation in wild-type mice. The result was highly reproduced between several animals. This orientation preference was less prominent in newborn mice, where glomerular vascularization is not fully developed, and totally lost in both genetic and non-genetic disease models with foot process effacement.

## Conclusions

Using novel imaging tools, we could, for the first time, establish that foot processes are not randomly distributed on glomerular capillaries. We have in previous studies suggested a role for foot processes to provide a counter-acting force to the capillary wall stress resulting from capillary blood pressure. If the capillary is approximated to have a cylindrical shape, the circumferential wall stress component is larger than the longitudinal wall stress component. Thus, the preferred orientation of foot processes in parallel with capillaries might be needed to withstand the higher wall stress component in this direction.

# Abstract: 9 Phosphorylation of ACTN4 leads to podocyte vulnerability and proteinuric glomerulosclerosis

**Authors:** Dr. Di Feng, Dr. Mukesh Kumar, Dr. Jan Muntel, Dr. Susan Gurley, Dr. Gabriel Birrane, Dr. Isaac Stillman, Dr. Lai Ding, Dr. Minxian Wang, Dr. Saima Ahmed, Dr. Johannes Schlondorff, Dr. Seth Alper, Dr. Tom Ferrante, Dr. Susan Marquez, Dr. Carlos Ng, Dr. Richard Novak, Dr. Donald Ingber, Dr. Hanno Steen, Dr. Martin Pollak

**Affiliations:** ^1^Beth Israel Deaconess Medical Center and Harvard Medical School, Boston, USA; ^2^Boston Children's Hospital, Boston, USA; ^3^Biognosys AG, Schlieren, USA; ^4^Division of Nephrology and Hypertension, Portland, USA; ^5^Wyss Institute, Boston, USA; ^6^NeuroTechnology Studio, Boston, USA; ^7^Broad Institute of Harvard and MIT, Boston, USA

## Background

Although genetic mutations in ACTN4—an important cytoskeletal protein—have been linked to proteinuric glomerulosclerosis in humans, the effect of post-translational modifications of ACNT4 on podocyte integrity and kidney function is not known.

## Methods

Using mass spectrometry, we found that ACTN4 is phosphorylated at S159. We used phosphomimetic and nonphosphorylatable ACTN4 to comprehensively study the effects of this phosphorylation *in vitro* and *in vivo.* We conducted x-ray crystallography, F-actin binding and bundling assays, and immunofluorescence staining to evaluate F-actin alignment. Microfluidic organ-on-a-chip technology was used to assess for podocyte detachment when simultaneously exposed to fluid flow and cyclic strain. We then used CRISPR/Cas9 to generate mouse models and assessed for renal injury by measuring albuminuria and examining kidney histology. We performed targeted mass spectrometry to determine whether high extracellular glucose and TGF-b increase phosphorylation of ACTN4.

## Results

Compared to wild type (WT), phosphomimetic ACTN4 demonstrated increased binding and bundling activity with F-actin *in vitro.* Phosphomimetic Actn4 mouse podocytes exhibited more spatially correlated F-actin alignment and a higher rate of detachment under mechanical stress. Phosphomimetic Actn4 mice developed proteinuria and glomerulosclerosis after subtotal nephrectomy. Moreover, we found that exposure to high extracellular glucose and TGF-b stimulates phosphorylation of ACTN4 at S159 in podocytes.

## Conclusions

These findings suggest that increased phosphorylation of ACTN4 at S159 leads to biochemical, cellular, and renal pathology that is similar to pathology resulting from human disease- causing mutations in ACTN4. ACTN4 may mediate podocyte injury as a consequence of both genetic mutations and signaling events that modulate phosphorylation.

# Abstract: 10 SMPDL3b regulates proteinuria without altering kidney function in experimental glomerular diseases

**Authors:** Alla Mitrofanova, Judith Molina, Javier Varona Santos, Matthew Tolerico, Antonio Fontanella, Lorenza Macrina, Professor George Burke, Associate Professor Sandra Merscher, Professor Alessia Fornoni

**Affiliations:** University of Miami, Miami, USA

Sphingosine-1-phosphate lyase insufficiency causes accumulation of sphingosine-1-phosphate (S1P) and is associated with focal segmental glomerulosclerosis, a renal disease that is often characterized by the recurrence of proteinuria after kidney transplantation. We previously reported a role of sphingomyelin phosphodiesterase acid-like 3b (SMPDL3b), which regulates lipid composition, in podocyte response to injury. In this study we tested the hypothesis that dysregulation of renal SMPDL3b expression affects the availability of S1P, thus contributing to proteinuria and chronic kidney disease (CKD) progression.

A murine model of Alport syndrome (AS), a surrogate model for proteinuric CKD, was used. AS mice were crossed to podocyte-specific Smpdl3b-deficient mice (DKO) and kidneys from 5-months-old animals were harvested and processed for in-depth phenotypical analysis, including urinary albumin-to- creatinine ratio (ACR), histological analysis, transmission electron microscopy analysis, glomeruli isolation and serum analysis. Lipidomic analysis was performed using LC-MS analysis. All animal studies were performed in accordance with the NIH IACUC Guidelines. For statistical analysis One-Way ANOVA or Student t-test were used. SMPDL3b expression was increased in kidneys of AS mice when compared to controls. We found decreased ACR in DKO mice compared to AS mice in association with increased S1P. Improved ACR was associated with less foot processes effacement, but was not associated with improved body weight, serum creatinine, glomerular filtration rate or renal fibrosis. DKO mice demonstrated significantly lower S1P levels in kidney cortexes compared to AS mice. Our data indicate that SMPDL3b expression may affect availability of S1P and regulate proteinuria without affecting disease progression in experimental proteinuric CKD.

# Abstract: 11 Characterization of Purinergic Receptor 2 Signaling in Diabetic Podocytes

**Authors:** Dr. Christine A. Klemens, Dr. Oleg Palygin, Dr. Elena Isaeva, Mr. Vladislav Levchenko, Dr. Denisha R. Spires, Dr. Lashodya Dissanayake, Ms. Oksana Nikolaienko, Dr. Daria V. Ilatovskaya, Dr. Alexander Staruschenko

**Affiliations:** ^1^Medical College of Wisconsin, Milwaukee, USA; ^2^Medical University of South Carolina, Charlestown, USA

Under normal conditions, extracellular ATP levels are low. However, extracellular ATP levels can increase dramatically during pathophysiological conditions, resulting in changes to purinergic receptor expression and signaling. The aim of this study was to characterize podocyte purinergic receptor 2 (P2) profile and intracellular calcium ([Ca^2^+]i) flux in response to ATP in type 1 and type 2 diabetic models with diabetic kidney disease. Type 2 diabetic nephropathy (T2DN) rats were compared to non-diabetic control (Wistar) and Goto-Kakizaki rats, which are diabetic but do not develop diabetic nephropathy. Streptozotocin-injected Dahl Salt-Sensitive rats were used as a type 1 diabetes model. Changes in [Ca^2^+]i flux in podocytes in *ex vivo* glomeruli from diabetic kidneys were measured using live confocal calcium imaging. A dramatic increase in the ATP-mediated [Ca^2^+]i response in podocytes was observed in both models of diabetes. Pharmacological inhibition established that, in contrast to healthy podocytes where purinergic signalling is predominantly mediated through metabotropic P2Y1 receptors, diabetic podocytes responded to ATP via ionotropic P2X4 and P2X7 receptors. Additionally, measurements of basal calcium concentrations in 12-week-old rats demonstrated that T2DN rats had significantly elevated basal calcium compared to Wistar rats, even before the development of albuminuria. The mRNA and protein expression profiles of P2 receptors in the kidney cortex revealed increased P2X4 and P2X7 receptor expression and decreased P2Y1 receptor abundance with diabetes. Therefore, P2 receptor remodelling from metabotropic to ionotropic may disrupt [Ca^2^+]i homeostasis in podocytes, resulting in their dysfunction and exacerbating the progression of diabetic kidney disease.

# Abstract: 12 Explainable Biomarkers for Automated Glomerular and Patient-Level Disease Classification

**Authors:** Mr. Matthew Basso, Dr. Moumita Barua, Dr. Rohan John, Dr. April Khademi

**Affiliations:** ^1^Image Analysis in Medicine Lab (IAMLAB), Department of Electrical, Computer, and Biomedical Engineering, Ryerson University, Toronto, Canada; ^2^Division of Nephrology, University Health Network, Toronto, Canada; ^3^Toronto General Hospital Research Institute, Toronto General Hospital, Toronto, Canada; ^4^Department of Medicine, University of Toronto, Toronto, Canada; ^5^Institute of Medical Sciences, University of Toronto, Toronto, Canada; ^6^Department of Pathology, UniversityHealth Network, Toronto, Canada; ^7^Image Analysis in Medicine Lab (IAMLAB), Department of Electrical, Computer, and Biomedical Engineering, Ryerson University, Toronto, Canada; ^8^Keenan research Center for Biomedcal Science, St. Michael's Hospital, Unity Health Network, Toronto, Canada; ^9^Institute for Biomedical Engineering, Science, and Technology (iBEST), a partnership between St. Michael's Hospital and Ryerson University, Toronto, Canada

## Background

Pathologists use multiple microscopy modalities to assess renal biopsies which is laborious, costly, has extensive turn-around times, and may not always be available. Computational approaches have potential to systematically quantitate subvisual clues to augment clinical workflows and evaluate linkages to clinical outcomes in large datasets for biomarker discovery. To this end, a proof of principle study is presented demonstrating that explainable biomarkers and machine learning can distinguish between three different glomerular disorders using PAS-stained images only.

## Methods

The proposed system extracts explainable biomarkers related to color, morphology, and microstructural texture and uses machine learning algorithms to automatically classify minimal change disease (MCD), membranous nephropathy (MN), and thin-basement nephropathy (TBMN). Disease labels are predicted on a per glomerular- and patient-level basis.

## Results

The final model combined the Gini feature importance set and Linear Discriminant Analysis classifier. Six morphological (ratio of nuclei-to-glomerular tuft area, ratio of nuclei-to-glomerular area, glomerular tuft thickness > 10, glomerular tuft thickness > 3, total glomerular tuft thickness, and glomerular circularity) and four microstructural texture features (luminal contrast using wavelets, nuclei energy using wavelets, nuclei variance using color vector LBP, and glomerular correlation using GLCM) were together the best performing features. Accuracy of 76.86% and 86.67% were obtained for glomerular and patient-level classification, respectively.

## Conclusion

The proposed image analysis algorithm measures explainable biomarkers to classify glomerular and predict patient-level classification. These biomarkers are compatible with our existing knowledge of disease pathogenesis and supports applying this algorithm to clinical datasets to identify novel prognostic and mechanistic biomarkers.

# Abstract: 13 Prothrombin Modulates Podocyte Health and Function During Glomerular Proteinuria

**Authors:** Dr Amanda Waller, Dr Katelyn Wolfgang, Dr Tasha Wilkie, Dr Sagar Bhayana, Dr William Smoyer, Dr Bryce Kerlin

**Affiliations:** ^1^The Research Institute at Nationwide Children's Hospital, Columbus, USA; ^2^The Ohio State University College of Medicine, Columbus, USA

## Background

Ongoing podocyte injury is a known critical determinant of glomerular disease progression. Recent research suggests thrombin exacerbates podocyte injury during proteinuria. However, pharmacologic thrombin inhibition may cause both on-and-off-target effects. The purpose of this study was to examine the effects of thrombin on podocyte health by manipulating its zymogen precursor, prothrombin (PT). We hypothesized that circulating PT would directly modulate in situ podocyte function, health, and survival in a rat model of glomerular proteinuria.

## Methods

Puromycin aminonucleoside (PAN)-induced proteinuria was treated with: (1) PT antisense oligonucleotide to induce hypoprothrombinemia (LoPT), (2) Serial i.v. PT infusions to sustain hyperprothrombinemia (HiPT), or (3) sham (PAN only) Controls (n=12/group). Morning spot urine and citrated plasma were collected at day 10. Plasma PT activity was measured by chromogenic assay. Glomeruli were isolated, dissociated into single-cell suspensions, and analyzed by flow cytometry after immunofluorescent synaptopodin antibody and TUNEL staining. Podocyte counts in whole glomeruli were confirmed using z-stacking confocal microscopy.

## Results

Compared to Control, LoPT reduced proteinuria and terminal podocyte injury (TUNEL-positive podocyte fraction), whereas HiPT worsened proteinuria and enhanced podocyte injury. Additionally, circulating PT levels modulated in situ podocyte preservation resulting in fewer podocytes per glomerulus in HiPT vs LoPT.

## Conclusion

Circulating prothrombin levels modulate podocyte function (proteinuria), health (terminal injury), and survival in the PAN model of glomerular proteinuria. Future studies should work to determine the prothrombinase mechanism that enables thrombin formation and signaling in the glomerulus and evaluate its potential as a novel therapeutic target to slow glomerular disease progression.

# Abstract: 14 Expression of PODXL and TRPC6 in podocyte cell culture after albumin overload with and without puromycin-aminoglycoside damage

**Authors:** Marcela Souza, Mariana Fontana, Adriana Torsoni, Vera Belangero, Andréa Maciel-Guerra, Gil Guerra-Júrior, Maricilda Mello, Mara Guaragna

**Affiliations:** University of Campinas, Sao Paulo, Campinas, Brazil

## Background

Proteinuria is considered a prognostic marker for kidney disease and one of the main symptoms of diseases affecting podocytes, the so called podocytopathies. Nevertheless, there is no conclusive understanding on the secondary effects of the protein flow on podocytes and how podocytes respond to this injury. Therefore, this study aimed to evaluate the expression of PODXL and TRPC6 genes after supplementation of albumin in podocytes in vitro. Methods: We exposed conditionally immortalized human podocytes to progressive concentrations of albumin (0, 3, 6, 9, 12, 15, 18, 20, 30 and 40 mg/ml) for 24 hours under two conditions: 1) without treatment and 2) after exposure to puromycin aminoglycoside (PAN). We evaluated PODXL and TRPC6 relative expression through qPCR. Statistical analyzes were performed with ANOVA and Tukey's a posteriori test (p<0.05). Results: We observed a higher rate of PODXL transcript in both groups (p<00001). On the other hand, TRPC6 presented a decrease in the transcript level after supplementation in the non-PAN group (p<00001) and an increase in the rate in the 40 mg/mL/PAN treatment group (p<00001). We suggest that PODXL overexpression in both groups may represent a defence mechanism of podocytes to maintain slit diaphragms intact. Conversely, TRPC6's expression diverged in each group. When considering the PAN group, the higher Ca+2 influx caused by TRPC6 increased expression may increase apoptosis, aggravating podocyte damage after proteinuria insult. Conclusions: This is a pilot study for future studies on molecular mechanisms in vivo that could be associated with podocyte damage caused by proteinuria.

# Abstract: 1 HGF-induced activation of Nephrin and Neph1 serves as a novel mechanism for recovery of podocytes from injury

**Authors:** Dr. Ashish Solanki, Dr. Pankaj Srivastava, Dr. Ehtesham Arif, Mrs. Bushra Rahman, Dr. Avinash Singh, Dr. Sang Ho Kwon, Dr. Matthew Lazzara, Dr. Joshua Lipschutz, Dr Christopher Davies, Dr. Deepak Nihalani

**Affiliations:** ^1^Medical University of South Carolina, Charleston, USA; ^2^Augusta University, Augusta, USA; ^3^University of Virginia, Charlottesville, USA

## Background

The extracellular domains of podocyte proteins NEPHRIN and NEPH1 serve as the building blocks for slit diaphragm, which are critical components of glomerular filtration barrier. Although these are signaling proteins, the activation mechanisms for these proteins have never been described. Here, we present a novel concept showing that apart from structural organization, NEPHRIN and NEPH1 constitute a receptor-based function, where these proteins can be activated in a ligand-induced fashion.

## Methods

Proteomics, Cloning, protein-expression and purification (using baculovirus-systems), SPR (surface- plasma-resonance), Co-Immunoprecipitation, ELISA, Immunofluorescence.

## Results

Since NEPHRIN and NEPH1 interacted with tyrosine phosphatase SHP-2 in a phosphorylation-dependent manner, we investigated whether ligands that induce SHP-2 stimulation also induced NEPHRIN and NEPH1 activation. Screening of multiple ligands identified HGF as a prominent inducer of NEPHRIN and NEPH1 phosphorylation. To establish HGF as a ligand, we used baculovirus expression system to generate purified NEPHRIN and NEPH1 proteins and confirmed a direct interaction between HGF and the extracellular domains of NEPHRIN and NEPH1. We also demonstrate ligand-induced phosphorylation of these proteins. Additionally, we demonstrate that SHP-2 directly dephosphorylates these proteins, thus presenting for the first time an activation and deactivation mechanism for these proteins. We further demonstrate that HGF-induced activation of these proteins is critical for podocytes recovery from injury, where addition of purified HGF to injured podocytes significantly enhanced their recovery in a NEPHRIN and NEPH1-dependent fashion. Conclusions: We provide compelling evidence that ligand-induced activation of NEPHRIN and NEPH1 by HGF is a component of podocyte recovery mechanism that restores lost podocyte function.

# Abstract: 2 FRET-based visualization of compartmentalized cGMP signalling in podocyte

**Authors:** Nelli Rutkowski, Dr. Frederik Görlitz, Dr. Martin Höhne, Prof. Dr. Thomas Benzing, Dr. Matthias Hackl

**Affiliations:** ^1^Department II of Internal Medicine and Center for Molecular Medicine Cologne, University of Cologne, Faculty of Medicine and University Hospital Cologne, Cologne, Germany; ^2^CECAD, University of Cologne, Faculty of Medicine and University Hospital Cologne, Cologne, Germany; ^3^Advanced Bioimaging Center, Department of Molecular and Cell Biology, University of California, Berkeley, USA

## Background

Cyclic guanosine 3',5'-monophosphate (cGMP) is an ubiquitous intracellular second-messenger and generated by nitric oxide (NO)/soluble guanylate cyclase (sGC) and natriuretic peptide (NP)/particulate guanylate cyclase (pGC) signalling cascades. Drugs targeting this pathway have been successful in the treatment of vascular disease and sparked interest in translation to renal disease. Nevertheless, the fundamental physiological mechanisms remain incompletely understood.

## Methods

We isolated viable glomeruli from mice expressing the genetically encoded fluorescent biosensor cGi- 500 exclusively in the cytosol of podocytes. Binding of free cGMP initiates a decrease in FRET efficiency, as evidenced by a simultaneous change of the donor and acceptor emission intensity in opposite directions. The donor/acceptor emission ratio (CFP/YFP) reflects the change in cGMP concentration over time. Agents modulating the cGMP signalling pathway were used together with a superfusion system and confocal microscopy. Furthermore, acute kidney slices were prepared and subsequently imaged for intensity and FLIM-FRET measurements with multiphoton microscopy.

## Results

While the synthesis of cGMP rapidly decreases after NO donor incubation in the wash phase, cGMP synthesis increases after NP incubation over the measurement period. This result suggests that the generated cGMP pools perform different tasks in different locations, since NO donors activate sGC in the cytosol and pGC operate on membrane-bound NP receptors.

## Conclusions

Podocytes are targeted by vasoactive substances and respond with spatiotemporal separability in the synthesis of cGMP after NO and NP incubation.

# Abstract: 3 Complementary Nck1 and Nck2 signaling in podocytes controls alpha actinin-4-mediated actin organization, adhesion and basement membrane composition

**Authors:** Dr. Claire Martin, Mr. Noah Phippen, Ms. Ava Keyvani Chahi, Dr. Manali Tilak, Dr. Sara Banerjee, Ms. Peihua Lu, Dr. Laura New, Mr. Matthew Platt, Dr. Mira Krendel, Dr. Jeremy Simpson, Dr. Nicolas Bisson, Dr. Anne-Claude Gingras, Dr. Nina Jones

**Affiliations:** ^1^Lunenfeld-Tanenbaum Research Institute, Sinai Health System, Toronto, Canada; ^2^Department of Molecular and Cellular Biology, University of Guelph, Guelph, Canada; ^3^Department of Biochemistry and Biomedical Sciences, McMaster University, Hamilton, Canada; ^4^Centre de recherche du Centre Hospitalier Universitaire (CHU) de Quebec-Université Laval, Québec, Canada; ^5^Centre de recherche sur le cancer de l'Université Laval, Québec, Canada; ^6^Human Health and Nutritional Sciences, University of Guelph, Guelph, Canada; ^7^Cell and Developmental Biology Department, State University of New York Upstate Medical University, Syracuse, USA; ^8^Department of Molecular Biology, Medical Biochemistry and Pathology, Université Laval, Québec, Canada; ^9^Department of Molecular Genetics, University of Toronto, Toronto, Canada

## Background

Maintenance of the kidney filtration barrier requires coordinated interactions between podocytes and the underlying glomerular basement membrane (GBM). GBM ligands bind podocyte integrins to trigger actin-based signaling events critical for adhesion. Nck1/2 adaptors have emerged as essential regulators of podocyte cytoskeletal dynamics; however, the precise mechanisms they mediate remain to be fully elucidated.

## Methods

Cultured podocyte cell and mouse models deficient in Nck1 and/or Nck2 were generated. Transcriptomic approaches were used to profile expression differences. Proteomic techniques were applied to identify specific binding partners for Nck1 and Nck2. Molecular manipulations of cells (CRISPR knockout and viral-mediated rescues) exposed signaling mechanisms by which Nck1/2 function, which were further validated *in vivo* using knockout and disease models.

## Results

Compound loss of Nck1/2 resulted in altered expression of genes involved in cell adhesion, actin binding and extracellular matrix composition. Accordingly, defective adhesion and actin organization were observed in Nck1/2 deficient podocytes *in vitro,* and podocyte detachment and altered GBM morphology was present *in vivo.* Distinct interactomes for Nck1 and Nck2 were identified, helping reveal a mechanism by which they cooperate to regulate actin bundling and focal adhesion sustenance through alpha actinin-4. Singular loss of (or partial rescue by) Nck1 or Nck2 caused intermediate disruptions *in vitro* and *in vivo,* resulting in increased matrix deposition and enhanced susceptibility to podocyte injury.

## Conclusions

These findings highlight distinct yet complementary roles for Nck1/2 in regulating podocyte adhesion *via* alpha actinin-4 and reveal novel functions for Nck1/2 in controlling GBM composition and sustaining filtration barrier integrity.

# Abstract: 4 Selective Modulation of Nuclear Receptor PPARy by a Partial Agonist with Reduced Adipogenic Potential Ameliorates Experimental Nephrotic Syndrome

**Authors:** Ms. Claire Burton, Mr. Galen Rask, Dr. Amanda Waller, Dr. Amy Webb, Dr. Marina Galdino-Pitta, Dr. Angelica Amato, Dr. Rachel Cianciolo, Dr. Brian Becknell, Dr. Bryce Kerlin, Dr. Francisco Neves, Dr. Alessia Fornoni, Dr. Shipra Agrawal

**Affiliations:** ^1^Abigail Wexner Research Institute at Nationwide Children's Hospital, Columbus, USA; ^2^The Ohio State University, Columbus, USA; ^3^Laboratory of Design and Drug Synthesis, Bioscience Center, Federal University of Pernambuco, Recife, Brazil; ^4^Laboratório de Farmacologia Molecular, Universidade de Brasilia, Brasilia, Brazil; ^5^University of Miami Miller School of Medicine, Miami, USA

## Background

Glomerular disease, often manifesting as nephrotic syndrome (NS) with high proteinuria, can be refractory to standard treatment and is typically associated with hypoalbuminemia, hypercholesterolemia, and hypercoagulopathy. We hypothesized that the nuclear receptor PPARg can be selectively modulated using a novel partial agonist, GQ-16, to gain therapeutic advantage over traditional PPARg agonists for NS treatment.

## Methods

Pio and GQ-16 were administered daily to male Wistar rats with puromycin amino-nucleoside (PAN)-induced nephropathy. Serum and urine chemistries were performed and kidneys, glomeruli, liver, and white adipose tissue (WAT) were harvested for RNA and protein extraction. Blood was collected for determination of thrombin generation parameters.

## Results

PAN induced robust proteinuria, which was significantly reduced with Pio to 64% of PAN-value, and robustly with GQ-16 to 81% of PAN, which was comparable to controls. Podocyte hypertrophy also returned to normal with Pio and GQ-16. While both GQ-16 and Pio restored glomerular *Nphs1* and hepatic *Pcsk9* expression and reduced hypercholesterolemia, GQ-16 also restored glomerular *Nrf2,* and reduced disease-associated hypoalbuminemia and hypercoagulopathy. Furthermore, RNA-seq analysis identified both common and distinct glomerular genes altered by Pio and GQ-16 treatments. Moreover, Pio but not GQ-16 significantly induced aP2 (fatty acid binding protein) in adipocytes and in WAT. Both, Pio and GQ-16 induced insulin sensitizing adipokines in WAT with varying degrees.

## Conclusions

Selective modulation of PPARg by a partial agonist may be a more beneficial approach than a full PPARg agonist in reducing proteinuria, hypoalbuminemia, and hypercoagulopathy in NS and in decreasing drug-associated side effects such as adipogenesis and weight gain.

# Abstract: 1 The Effect of Insulin on Lysosome Activity in Cultured Rat Podocytes

**Authors:** Patrycja Rachubik, Irena Audzeyenka, Magdalena Wysocka, Marlena Typiak, Dorota Rogacka, Prof. Adam Lesner, Agnieszka Piwkowska

**Affiliations:** ^1^Laboratory of Molecular and Cellular Nephrology, Mossakowski Medical Research Institute, Polish Academy of Sciences, Gdansk, Poland; ^2^Faculty of Chemistry, University of Gdansk, Gdansk, Poland

Insulin is a key regulator of podocyte function and morphology. However, a long-term exposure of podocytes to insulin induces insulin resistance by lysosomal degradation of the insulin receptor, which suggests an important role of lysosomes in the regulation of insulin signalling pathway. The direct effect of insulin on the lysosomes' activity in cultured rat podocytes is unknown, therefore we investigated the influence of insulin on the expression of LAMP1 and cathepsin D (CTPD), i.e. markers of lysosomes.

The effect of insulin (100 nM) on the mRNA (qPCR), total LAMP1, proCTPD, CTPD protein expressions (Western blot), and CTPD activity (a fluorescent substrate of CTPD) was measured. In order to determine the impact of insulin on the CTPD and LAMP1 localization in podocytes immunofluorescence staining was performed. The LysoTracker® was used to label lysosomes in insulin-treated podocytes.

A short-term incubation of podocytes with insulin decreased the mRNA expression and total LAMP1 and CTPD protein expressions, whereas the proCTPD protein level increased. The incubation of podocytes with insulin significantly reduced the CTPD activity - by 27% (0.93±0.08 vs. 0.68±0.05, n=8, P < 0.05) after 3 min and by 44% (0.93±0.08 vs. 0.52±0.07, n=8, P < 0.001) after 15 min. The usage of bafilomycin A1 decreased CTPD activity and protein levels of CTPD and LAMP1.

These results suggest that insulin regulates lysosome activity in podocytes. The state of insulin resistance may impair podocyte function as a consequence of the altered lysosomal cathepsin D activity.

This work was supported by the National Science Centre (grant number 2018/29/N/NZ4/02212).

# Abstract: 2 PTEN-induced kinase 1 (PINK1) deficiency impairs insulin signaling pathway in human podocytes.

**Authors:** Dr Irena Audzeyenka, Miss Patrycja Rachubik, Dr Marlena Typiak, Mr Tomasz Kulesza, Miss Daria Kalkowska, Dr Dorota Rogacka, Prof Stefan Angielski, Prof Agnieszka Piwkowska

**Affiliations:** Mossakowski Medical Research Centre PAS, Gdansk, Poland

Diabetic environment induces changes in podocyte bioenergetics, which is tightly linked with mitochondrial dynamics and mitophagy. Mitophagy is a process of selective degradation of non- functional or damaged mitochondria. The main goal of this study was to elucidate the role of PINK1- dependent mitophagy in podocyte biology and function.

In order to inhibit mitophagy, we generated a human podocyte cell line stably expressing PINK1 shRNA through lentiviral transduction. The expression of mRNAs and proteins were determined using real-time PCR and western blot. Membrane fractions of proteins were quantified after cell biotinylation. Albumin permeability was evaluated by measuring FITC-labelled BSA diffusion across a podocyte monolayer. Glucose transport was assessed by measurements of [12-3H]-deoxy-D-glucose uptake. Immunofluorescence was used to track intracellular localization of proteins.

We observed a 19% decrease in podocin expression in PINK1-depleted podocytes, with comparable levels of nephrin. However, membrane fractions of both proteins were significantly lower compared with control cells: by 47% for podocin and by 42% for nephrin. This was accompanied with 36% increase in albumin permeability in shPINK1 expressing podocytes. PINK1 inhibition had considerable effect on the membrane expression of insulin receptor and glucose transporter type 4 (GLUT4), which were reduced by 75% and 50%, respectively. Consequently, PINK1-depleted podocytes exhibited a significantly reduced glucose uptake.

These findings confirm an important role of mitophagy in the podocytes biology and provide a novel insight in the molecular mechanism which may underlie the development of podocyte insulin resistance.

This work was supported by grant 2016/23/D/NZ5/01449 from the National Science Centre, Poland.

# Abstract: 3 Low sensitivity to ATP protects podocytes from calcium transients

**Authors:** Dr. Julia Binz-Lotter, Eva Wiesner, Prof. Dr. med. Thomas Benzing, Dr. med. Matthias Hackl

**Affiliations:** ^1^Department II of Internal Medicine and Center for Molecular Medicine Cologne, University of Cologne, Faculty of Medicine and University Hospital Cologne, Cologne, Germany

## Background

ATP signaling is involved in a plethora of pathways, involving damage signaling. Cell culture models as well as experiments on isolated glomeruli indicate that podocytes respond to ATP with a calcium transient. To date a direct effect of ATP on podocyte calcium levels has not been demonstrated *in vivo*.

## Methods

In mice expressing GCaMP3 in podocytes (control vs. mice treated with nephrotoxic serum) or in endothelial cells *ex vivo* calcium imaging of acute kidney slices (AKS) and 2-photon *in vivo* calcium imaging was carried out. AKS were stimulated with ATP and intracellular calcium levels recorded. For *in vivo* imaging, mice were anaesthetized, an arterial catheter was placed into the right carotid artery or the aorta and the left kidney was exteriorized. During *in vivo* imaging, different doses of ATP were injected as a bolus via the catheter and calcium transients were monitored.

## Results

Stimulation with ATP resulted in robust calcium transients in glomerular endothelial cells *ex vivo* and *in vivo*, while even a 5- to 10-fold increase of the ATP concentration did not trigger any calcium transients in podocytes neither *ex vivo* nor *in vivo*. After the induction of podocyte injury by the injection of nephrotoxic serum, podocytes responded with similar calcium transients as endothelial cells.

## Conclusions

In contrast to endothelial cells, only injured podocytes respond to ATP with calcium transients. We therefore hypothesize, that the low sensitivity ATP of healthy podocytes is a protection mechanism to avoid possible deleterious calcium signals from filtered ATP.

# Abstract: 4 *Drosophila* Filamin exhibits a mechano-protective role during nephrocyte injury via hypertrophy

**Authors:** Dr Sybille Koehler, Dr Barry Denholm Biomedical

**Affiliations:** Sciences, University of Edinburgh, Edinburgh, United Kingdom

## Background

Podocytes are constantly exposed to biomechanical forces such as shear stress and hydrostatic pressure. These forces increase during disease like diabetes and hypertension, resulting in adaptive mechanisms such as podocyte hypertrophy. But how do podocytes sense changes in biomechanical forces and how does the molecular response look like?

## Methods

To investigate this, we utilized *Drosophila* nephrocytes and studied the functional role of the mechanosensor Cheerio (dFilamin). FilaminB is upregulated in podocyte injury models and upon increased biomechanical stress, therefore serving as an ideal candidate for mediating mechano- protection in response to injury.

## Results

Expression of an over-active mechanosensor region variant of Cheerio resulted in a significant hypertrophy phenotype, while morphology and filtration function were only mildly affected. Interestingly, the expression of over-active Cheerio caused a rescue of filtration function after depletion of the nephrocyte diaphragm proteins Duf (dNEPH) and Sns (dNephrin). Additional analysis with human FilaminB confirmed this mechano-protective role and the involvement of the mechanosensor region in the hypertrophy phenotype. To delineate the mechano-protective pathway acting downstream to Cheerio we studied the candidates: TOR, WNT and YAP. Activation of these pathways result in nephrocyte hypertrophy. Interestingly, TOR repression reversed the hypertrophy in over-active Cheerio expressing cells, suggesting TOR to be a novel downstream target of Cheerio and to be responsible for the hypertrophy phenotype.

## Conclusions

Although Cheerio and FilaminB mediate a mechano-protective role in the face of injury, their excessive expression resulted in a severe morphological and functional phenotype, emphasizing the need of a tight control of expression levels.

# Abstract: 5 Podocyte nephronectin expression is tightly controlled by non-canonical TGFβ pathways

**Authors:** Dr. rer. nat. Nina Sopel, Alexandra Ohs, Prof. Dr. med. Mario Schiffer, Prof. Dr. med. Janina Müller- Deile

**Affiliations:** Universitätsklinikum Erlangen - Department of Medicine 4 - Nephrology and Hypertension, Erlangen, Germany

## Background

Glomerular endothelial cells (GEC), together with podocytes and the glomerular basement membrane (GBM) build the glomerular filtration barrier. The components of the extracellular matrix (ECM) of the GBM are mainly produced by GECs and podocytes. Nephronectin (NPNT), which is mainly expressed by podocytes, is one important ECM protein for GBM integrity and has been shown to be altered in different kidney diseases. While NPNT was reported to be increased in diabetic nephropathy, NPNT levels in membranous glomerulonephropathy and FSGS were reduced. Furthermore, NPNT has been shown to be down-regulated by transforming growth factor beta (TGFP), on both mRNA and protein level.

## Methods

To further investigate the pathways that mediate this down-regulation, NPNT expression was analyzed in the established model of immortalized human podocytes. Differentiated cells were pre-incubated with inhibitors of different components of the non-canonical and the canonical TGFp pathway and subsequently cultured in the presence/absence of recombinant TGFp. Cell culture supernatants were subjected to ELISA and cell lysates used for qPCR or Western Blot analyses.

## Results

While pre-treatment with TGFβ-pathway inhibitors did not alter NPNT secretion, it was observed that NPNT expression was regulated post-transcriptionally, as no difference in *NPNT* mRNA levels were noted. Furthermore, on protein level under baseline conditions NPNT expression was increased after inhibition of non-canonical pathway components and additional treatment with TGFp had no further influence.

## Conclusions

Based on our results, we propose that NPNT expression in podocytes is post-transcriptionally fine-tuned, predominantly by the non-canonical TGFp pathway.

# Abstract: 6 The mechano-sensitive ion channel Piezo activates Rho1 in *Drosophila* nephrocytes

**Authors:** Dr Sybille Koehler, Dr Barry Denholm Biomedical

**Affiliations:** Sciences, University of Edinburgh, Edinburgh, United Kingdom

## Background

Podocytes constantly face biomechanical forces such as shear stress and hydrostatic pressure. Increasing forces result in morphological changes, detachment from the glomerular basement membrane and loss into the primary urine. This highlights a requirement for podocytes to sense changes in their physical environment and induce a response to react to increased biomechanical force.

## Methods

Here, we investigated the functional role of the mechano-sensitive ion channel Piezo in *Drosophila* nephrocytes.

## Results

First, we confirmed Piezo expression and localisation at the nephrocyte diaphragm. Acute activation of the channel with the chemical compound YODA revealed significantly increased Ca++ signalling and Rho1 activation, suggesting a functional role of Piezo in nephrocytes and delineating the putative Piezo mechanosensitive pathway.

For further analysis, we used knockout flies and observed a filtration phenotype, while morphology and GTPase activation was not altered. In addition, we also studied the impact of elevated Piezo levels and could show, that in line with the YODA effect, Piezo overexpression revealed severely increased Rho1- GTP levels and FITC uptake, while morphology was not changed. Because of this severe pathological phenotype, we tried to rescue the effects of Piezo overexpression with pharmacological inhibition by using tarantula toxin. Intriguingly, treatment with tarantula toxin reversed the elevated Rho1-GTP levels observed upon Piezo overexpression.

## Conclusions

Taken together, our data confirms the functional expression of Piezo in nephrocytes, its role in regulating GTPases and the beneficial effect of tarantula toxin to reverse the pathological effects caused by increased Piezo levels.

# Abstract: 7 Doxorubicin toxicity in podocytes is associated with phenomena of mitotic catastrophe with accelerated senescence, autophagy and reticulum stress.

**Authors:** Doctor Hilal Yeter-Alat, Doctor Hans-Kristian Lorenzo

**Affiliations:** ^1^INSERM U1197, Villejuif, France; ^2^Bicetre Hospital/Department of Nephrology, Villejuif, France; ^3^University of Paris-Saclay, Villejuif, France

## Background

Podocytes are highly differentiated epithelial cells essential in renal filtration. They are postmitotic cells and are not replaced after death, so morphological alterations and their disappearance have serious consequences, especially proteinuria and nephrotic syndrome. Despite numerous studies, there is no consensus on the mechanisms of podocyte cell death.

## Methods

Doxorubicin (DOXO) is often used as an animal model of proteinuria. Here we used DOXO in vitro to induce stress in podocytes and examine morphological and functional alterations associated with cell death.

## Results

DOXO at high doses causes podocyte mortality. However, it does not seem to be justified by caspase- dependent apoptosis, since only a slight cleavage of caspase-3 was observed. Also, cellular hypertrophy associated with polyploidy and frequent nuclear morphological aberrations was observed.

The appearance of markers of accelerated senescence (b-gal) was detected and, at high doses, there was disappearance of p21 expression. The overexpression of several G2 checkpoint genes, such as ATM, ATR, Chk1, Chk2, followed by structural changes of centrin or lamin B suggest the presence of mitotic catastrophe induced by doxorubicin-induced DNA damage. In addition, we observed signs of endoplasmic reticulum stress (cleaved ATF6, p-IRE1, p-JNK) and autophagy (ATG3, ATG7, LC3I/II).

## Conclusions

Doxorubicin, widely used as a model for nephrotic syndrome, appears to induce podocyte death through mechanisms resulting from DNA damage including senescence, mitotic catastrophe involving autophagy and reticulum stress. These phenomena probably justified the deadly fate of podocytes since the involvement of caspases in this model appears residual.

# Abstract: 8 Divergent Impacts of Nck1 and Nck2 Loss on Diabetic Podocytopathy

**Authors:** Ms. Casey R. Williamson, Ms. Peihua Lu, Ms. Laura A. New, Dr. Nina Jones

**Affiliations:** Department of Molecular & Cellular Biology, University of Guelph, Guelph, Canada

Diabetic podocytopathy (DP) is a life-threatening kidney complication that involves damage to the podocyte slit diaphragm (SD). Nck adaptor proteins are critical for podocyte morphology and SD function. In patients with diabetes, increased *NCK1* mRNA, but decreased *NCK2,* is associated with diabetic nephropathy (DN). Mice with systemic knockout (KO) of either Nck1 or Nck2 show no overt alterations of podocyte function, but it is possible these mice exhibit altered susceptibility to DP. Therefore, this study aims to examine the degree of DP injury in diabetic Nck1 and Nck2 KO mice.

8-week-old male WT, Nck1KO, and Nck2KO mice were either administered streptozotocin (STZ) for diabetes induction, or citrate buffer as a respective control (n=4 per genotype per condition). Proteinuria was assessed by albumin excretion rate using metabolic cage urine collections. 17 weeks after STZ injection, tissues were processed for serology, renal cortical mRNA quantification, glomerular protein analysis, periodic acid-Schiff staining, and immunofluorescence.

Nck2KO-STZ mice exhibited greater proteinuria than diabetic WT- and Nck1KO-STZ mice. Histologically, podocyte density was equivalently reduced in all three diabetic groups, and surprisingly, glomerulosclerosis and glomerular hypertrophy were increased in WT-STZ, but not in either of the NckKO-STZ mice. Serum creatinine, cortical vimentin mRNA, and total nephrin protein (markers for glomerular filtration rate, epithelial-mesenchymal transition, and SD structure, respectively), were significantly elevated in Nck2KO-STZ, but not in WTs-STZs and Nck1KO-STZs, reflecting our findings regarding proteinuria.

In agreement with human datasets, this study shows that a loss of Nck2, but not Nck1, is associated with increased DP/DN susceptibility.

# Abstract: 9 Dok1/2 adaptor proteins regulate adhesion in kidney podocytes

**Authors:** Ms. Nikkita Dutta, Mr. Noah Phippen, Dr. Claire Martin, Ms. Peihua Lu, Mr. Lamine Aoudjit, Dr. Laura New, Dr. Tomoko Takano, Dr. Nina Jones

**Affiliations:** ^1^University of Guelph, Guelph, Canada; ^2^Lunenfeld-Tanenbaum Research Institute, Toronto, Canada; ^3^McGill University, Montreal, Canada

## Background

Podocyte adhesion to the underlying glomerular basement membrane is required for proper glomerular filtration, and this is mediated by dynamic signaling events within focal adhesions. Integrins are a major component of focal adhesions, and their activity and matrix binding is negatively regulated by the adaptor proteins Dok1 and Dok2. We identified Dok1 as an Nck binding partner expressed in the glomerulus, and herein investigated the role of Dok1/2 in podocytes.

## Methods

We evaluated the renal phenotype of Dok1/2 total body knockout (KO) mice and tested their response to podocyte injury using the nephrotoxic serum (NTS) model. In parallel, we generated Dok1/2 KO podocyte lines for use in functional assays. Lastly, we assessed expression of Dok1/2 in models of kidney injury.

## Results

Loss of Dok1/2 does not impact glomerular function with aging. However, Dok1/2 KO mice produce an attenuated response to podocyte injury. We further show that glomeruli from Dok1/2 KO animals display higher surface levels of integrin β1 relative to WT controls with a concomitant increase in active integrin β1, and that phosphorylation of the focal adhesion protein paxillin is significantly increased. In primary podocytes, we observe enhanced paxillin levels in focal adhesions in Dok1/2 KO cells, and Dok1/2 KO podocytes generated by CRISPR-Cas9 genome editing are more adhesive than control cells. Finally, we demonstrate that Dok1 levels are increased following injury both *in vitro* and in glomeruli of PAN-treated rats.

## Conclusion

Dok1/2 regulate integrin signaling in podocytes, which may have implications in adhesion and maintenance of glomerular filtration.

# Abstract: 10 Recurrent nephrotic plasma activates pro-fibrotic signalling pathways downstream of protease-activated receptor 1

**Authors:** Miss Musleeha Chesor, Dr. Jack Tuffin, Dr. Carl May, Miss Irene Ghobrial, Professor Melissa Little, Professor Moin Saleem, Dr. Gavin Welsh

**Affiliations:** ^1^University of Bristol, Bristol, United Kingdom; ^2^Princess of Naradhiwas University, Narathiwat, Thailand; ^3^Murdoch Children's Research Institute, Melbourne, Australia; ^4^University of Melbourne, Melbourne, Australia

## Background

Post-transplant recurrence of steroid-resistant nephrotic syndrome (SRNS) is thought to be due to an unknown “circulating factor”, the identity of which has so far remained elusive. Our previous work suggests a signalling role for protease-activated receptor-1 (PAR-1), leading to impaired podocyte function. The signalling pathways downstream of PAR-1 in podocytes are unknown and could reveal novel mechanistic insights into the disease. Methods: Conditionally immortalized human podocytes (ciPods) were treated with PAR-1 agonist peptide or nephrotic plasma (NP), in the absence or presence of four different PAR-1 antagonists.

## Results

PAR-1 agonist and patient relapse NP, but not paired remission plasma, induced the phosphorylation of VASP, JNK, and proteins involved in pro-fibrotic pathways. These changes were inhibited by PAR-1 inhibitors, but not by TGF-β1 inhibition. Four PAR-1 inhibitors demonstrated specific antagonistic properties. The phosphorylation of VASP and JNK in a new 3D spheroid model and from stem cell-derived kidney organoids corroborated the finding from the 2D model. Functionally, relapse NP induced podocyte motility and podocyte loss from spheroids both of which were also selectively rescued by PAR-1 inhibitors. Treatment of kidney organoids with relapse NP induced the same VASP and pro-fibrotic phosphorylation in podocytes and the loss of podocyte-specific markers.

## Conclusions

We propose that the circulating factor acts as a pro-fibrotic effector by activating PAR-1. A greater understanding of these signalling pathways will lead to the identification of novel therapeutic targets for this disease.

# Abstract: 11 SARA in Podocytes: Regulation of cell phenotype as a potential therapeutic target in glomerulosclerosis

**Authors:** Dr. Vidhi Dalal, Ms. Xiaoyan Liang, Dr. Tomoko Hayashida

**Affiliations:** Northwestern University, Chicago, USA

## Background

Podocytes play a central role in the development and progression of glomerulopathies. Injury to podocytes leads to their dedifferentiation, whereby the expression of podocyte-specific proteins is suppressed, and the expression of mesenchymal-specific proteins is induced. The consequent podocyte foot process effacement and detachment of podocytes from the glomerular basement membrane result in glomerulosclerosis. Our laboratory has identified Smad Anchor for Receptor Activation (SARA) as a key protein for maintaining cellular phenotype in the face of fibrogenesis. Here, we aim to determine if SARA overexpression in podocytes can prevent their dedifferentiation and protect against glomerulosclerosis.

## Methods

SARA^Tg^, podocin-Cre mice were used to overexpress SARA selectively in podocytes (SARA^podo^). SARA^Tg^ negative, podocin-Cre positive mice (Ctrl^podo^) were used as controls. The mice were treated with a one-time tail vein injection of Adriamycin to induce glomerulosclerosis. Urine, blood, and kidneys were harvested 3 to 14 days after injection for histological and molecular analyses.

## Results

SARA^podo^ mice showed less glomerulosclerosis histologically and less proteinuria than Ctrl^podo^ mice after Adriamycin treatment. Expression levels of markers specific for podocytes are being measured to confirm that SARA maintained cellular phenotype.

Gene expression profiles of podocytes isolated from SARA/Ctrl^podo^ mice by flow cytometry are being analyzed by RNA sequencing. This high throughput analysis will provide insight into the mechanisms by which SARA maintains podocyte phenotype and protects against glomerulosclerosis.

## Conclusions

SARA overexpression in podocytes protects against glomerulosclerosis. Elucidating the mechanisms by which SARA functions will help unearth new molecular targets for therapies directed at glomerular diseases.

# Abstract: 12 A novel proximity-dependent biotin identification (BioID) mouse model to identify the slit diaphragm proteome in health, aging, and disease

**Authors:** Dr Gary Gerlach, Dr Lori O'Brien

**Affiliations:** University of North Carolina at Chapel Hill, Chapel Hill, USA

Maintenance of podocyte foot process integrity is vital for kidney function. In particular, the slit diaphragm plays a critical role in supporting podocyte architecture and the glomerular filtration barrier. Despite the significance of this specialized intercellular junction, we lack a thorough understanding of how components of the slit diaphragm are modified with aging and disease. To begin addressing this gap, we developed a novel mouse model to identify the local slit diaphragm proteome. We modified the Nphs2 (podocin) locus to produce a podocin-BioID fusion protein. The promiscuous biotin ligase appended to podocin allows for unbiased biotinylation of proteins within proximity of the slit diaphragm. Strong, weak, and transient interactors are all captured by subsequent streptavidin isolation and mass spectrometry. We carried out our studies in mice aged 8 weeks and 2 years to identify age- related changes. We validated the proper localization and function of the podocin-BioID fusion by immunofluorescence, electron microscopy, and the generation of biotinylated proteins within podocytes. Mass spectrometry and analysis of the of the isolated proteome from 8-week-old mice identified ‘cell junctions’ and ‘adhesion’ as top gene ontogeny terms and included known slit diaphragm components. Additionally, we uncovered novel proteins such as immunoglobulin-like domain-containing receptor 2 (Ildr2) and we are currently assessing its role in podocyte function. Preliminary analysis of the 2-year proteome identified a decrease in Tensin2 (Tns2) which is important for podocyte-GBM interaction. Taken together, our studies have identified novel podocyte slit diaphragm interactors and will enable the identification of new biomarkers for kidney disease.

# Abstract: Defining Cluster Specificity of TNF Activation in Patients With Minimal Change Disease and Focal Segmental Glomerulosclerosis

**Authors:** Dr. Fadhl Alakwaa, Dr. Phillip J. McCown, Dr. Jennifer Harder, Mrs. Jamal El Saghir, Dr. Wenjun Ju, Dr. Matthias Kretzler, Dr. Laura H. Mariani, Dr. Sean Eddy

**Affiliations:** Michigan Medicine, Ann Arbor, USA

## Background

Mechanistic, targeted therapies are needed for patients with FSGS and MCD, as diverse biological processes produce similar histologic injury patterns.

## Methods

Consensus clustering was applied to bulk RNA sequencing (RNAseq) data from the glomerular (glom) and tubulointerstitial (TI) compartments of 231 participants from the NEPTUNE cohort. Clusters were assessed for association with clinical outcome. Differential gene expression analysis was analyzed for biological function between patient clusters. Single nuclear RNAseq was performed on patient samples to characterize cluster-specific gene profiles.

## Results

One cluster was associated with a higher risk of loss of kidney function over time and associated with TNF activation that was correlated across glom and TI compartments (r=0.76, p<0.0001). Single nuclear RNAseq from 10 NEPTUNE biopsies resulted in 45175 pooled nuclei and 15 clusters, which included all major kidney cell types. TNF expression was found in nuclei from immune clusters and in a proximal tubule and loop of Henle cluster. TNFRSF1A was universally expressed across cell clusters, while TNFRSF1B showed more restrictive expression. TNF target markers, CCL2 and TIMP1, demonstrated broad expression across many clusters including relatively high expression in podocytes. Kidney organoids confirmed MCP1 (encoded by CCL2) and TIMP1 upregulation by TNF treatment.

## Conclusion

TNF, TNF receptors, and TNF-responsive biomarkers reflect alterations in inflammatory and intrinsic kidney cell populations in patients with a TNF-associated signaling profile and are currently assessed as TNF target engagement biomarkers in a clinical trial of FSGS patients (NCT04009668).

# Abstract: 1 Genetic regulation of slit diaphragm formation and maintenance

**Authors:** Dr. Pei Wen, Dr. Jin-gu Lee, Dr. Jun-yi Zhu, Dr. Joyce van de Leemput, Dr. Zhe Han

**Affiliations:** Center for Precision Disease Modeling, Department of Medicine, University of Maryland School of Medicine, Baltimore, USA

## Background

The slit diaphragm is the most important cellular feature for podocyte function, but its formation and maintenance remains largely unknown. Progress has been hampered by challenges in accessibility and genetic manipulation of mammalian podocytes. *Drosophila* nephrocytes have a highly similar slit diaphragm structure that is much more accessible and easier to modified genetically.

## Methods

We leveraged Drosophila to decode the mysteries of how slit diaphragm is formed and maintained. We identified new markers for slit diaphragm in nephrocytes, and used these markers to track changes in the slit diaphragm in a systematic genetic screen in *Drosophila* nephrocytes to identify all genes required for slit diaphragm development and function. Results: We identified over 80 genes essential for slit diaphragm formation and maintenance. Many of them are involved in cell trafficking. These cell trafficking genes can be further characterized into categories involving a series of steps, including receptor-mediated endocytosis, vesicle sorting, vesicle fusion and exocytosis. We also identified cell trafficking pathways and genes that are dispensable for nephrocyte function.

## Conclusions

We identified cell trafficking genes that are essential for slit diaphragm filtration structure formation and maintenance in nephrocytes. Since all of these proteins are highly conserved from flies to humans, it is likely the same set of genes regulate podocyte slit diaphragm formation and maintenance. Mutations in these genes could also lead to genetic kidney diseases.

# Abstract: 2 The circadian regulation of glomerular function

**Authors:** Dr Rebecca Preston, Professor Qing-Jun Meng, Professor Rachel Lennon

**Affiliations:** University of Manchester, Manchester, United Kingdom

## Background

Peripheral tissues demonstrate circadian rhythmicity and dysfunction of core clock components has been implicated in the pathogenesis of many disease processes, including renal tubular dysfunction. However, the role of the circadian clock in the regulation of glomerular function is lacking. Glomerular disease is characterised by pathological proteinuria, which along with the rate of glomerular filtration, display robust circadian oscillation. It is therefore likely that an intra-glomerular clock is responsible for circadian oscillation in glomerular function.

## Methods

Circadian rhythms of gene expression were examined using real-time bioluminescence recordings of PER2::Luciferase reporter mice and human podocytes transfected with clock reporters. Using a mouse model expressing a knock-in fluorescent fusion of BMAL1, a core component of the molecular clock, quantitative live imaging was used to detect endogenous BMAL1. Global patterns of glomerular circadian gene expression were defined using transcriptional profiling of glomeruli isolated from wildtype mice over a circadian time-course.

## Results

Autonomous circadian oscillation was detected in glomeruli and podocyte cells for the first time. Quantitative live imaging confirmed high molecular abundance and nuclear-cytoplasmic mobility of endogenous BMAL1 in glomeruli and podocyte cells. Transcriptional profiling identified 260 glomerular rhythmic genes under circadian control and pathway analysis revealed genes implicated in glomerular physiology.

## Conclusions

Here we confirm the presence of intra-glomerular clock machinery and have identified the first glomerular circadian transcriptome. These results provide a firm basis for future studies that aim to elucidate the functional implication and therapeutic potential of circadian rhythm in glomerular health and disease.

# Abstract: 3 Developing targeted therapies for nephrotic syndrome in public private partnerships

**Authors:** Dr. Heather Ascani, Dr. Laura Mariani, Dr. Sean Eddy, Dr. Wenjun Ju, Ms. Hailey Desmond, Dr. Deb Gipson, Ms. Tina Mainieri, Dr. Matthias Kretzler

**Affiliations:** Nephrotic Syndrome Study Network (NEPTUNE) University of Michigan, Ann Arbor, Michigan, USA

## Background

Integrating public and private research efforts is key to expediting precision medicine for glomerular diseases. The Nephrotic Syndrome Study Network (NEPTUNE) has developed an innovative research and business model: “MATCH”, to enable targeted patient enrollment in clinical trials via the NEPTUNE public private partnership (NP5).

## Methods

The NEPTUNE community's knowledge base is leveraged to identify a molecular signal matching therapeutic targets under development by NP5 industry partners. The three steps of MATCH are: (1) predictive biomarker development; (2) patient communication of molecular information; and (3) return of results from NP5 partner clinical trials for evaluation of patient stratification effects on trial outcomes.

## Results

MATCH begins with NEPTUNE patient education on risks and benefits of the study. Once enrolled in MATCH, a patient's biosamples undergo biomarker profiling. MATCH provides individual pathway activation information to patients for consideration in their independent decision to enroll in a trial that may be more tailored to their specific disease and stage. Finally, clinical trial outcomes will be reported by NP5 partners to NEPTUNE to evaluate the MATCH impact on treatment responses.

## Conclusions

MATCH aims to test precision medicine across multiple trials and partners to move science closer to the overall goal: reaching the right patient with the right medicine at the right time. MATCH is an open framework available to additional partners interested in joining NP5.

# Abstract: 4 Syncytiotrophoblast Extracellular Vesicles Can Be Internalized By Human Podocytes And Influence Their Physiological Functions

**Authors:** Miss Shuhan Jiang, Dr. Toluwalase Awoyemi, Dr. Prassana Logenthiran, Miss Maryam Rahbar, Dr. Wei Zhang, Dr. Jack Tuffin, Prof. Moin Saleem, Prof. Manu Vatish

**Affiliations:** ^1^University of Oxford, Oxford, United Kingdom; ^2^University of Bristol, Bristol, United Kingdom

## Background

Circulating levels of syncytiotrophoblast extracellular vesicles (STBEVs) increase throughout gestation in pregnant women. During pregnancy, renal glomerular filtration rates increase by 50% which implicated the possible unregulated function of renal filtration barrier. The aim of this study is to evaluate whether STBEVs can be internalized by human podocytes and influence their functions.

## Methods

Small STBEVs (known as STB exosomes, STBEXs) were isolated from dual-lobe placental perfusion of normal pregnancy women (n=3) by ultracentrifuge and characterized by immunoblotting and nanoparticle tracking analysis. STBEXs at two different concentrations (20mg/ml and 40mg/ml) were stained with PKH26 and incubated with podocyte over a time course (15mins, 30mins, 2h, 4h, 6h and 24h). STBEXs internalization was assessed by flow cytometry and confocal microscopy. The nephrin expression and migration ability of podocytes influenced by STBEXs were assessed by western blot and migration assay.

## Results

The internalization of STBEXs by podocyte can be observed from 30mins and increased throughout the time course, which yield the highest signal at 24h incubation. Confocal microscopy supported STBEXs internalization into podocytes. The expression of nephrin on podocyte was increased by 2.4-fold after 30mins STBEXs incubation (p<0.05). The migration ability of podocytes stimulated with STBEXs was upregulated by 1.2-fold (p<0.05) over 14-hours' observation.

## Conclusions

STBEXs can be taken up by podocytes and alter podocytes' functions through alternating nephrin expression and enhancing podocyte migration ability. We speculate one of the functions STBEXs on podocyte is enhancing the slit diaphragm structure and may contribute to the higher filtration rate seen in pregnancy.

# Abstract: 5 Glucocorticoid- and Pioglitazone-Induced Proteinuria Reduction Correlates with Glomerular Extracellular Matrix Remodeling in Experimental Nephrotic Syndrome

**Authors:** Dr. Sagar Bhayana, Dr. Shipra Agrawal, Ms. Melinda Chanley, Dr. Amanda Waller, Ms Katelyn Wolfgang, Dr. Saranga Wijeratne, Dr. James Fitch, Dr. Peter White, Dr. Bryce Kerlin, Dr. William Smoyer

**Affiliations:** Nationwide Children's Hospital, Columbus, USA

## Background

Nephrotic Syndrome (NS) is a common glomerular disease in children. While glucocorticoids (GC) are the primary treatment, pioglitazone (Pio; an FDA-approved PPARg agonist to treat type 2 diabetes) is known to both reduce proteinuria in experimental NS and directly protect podocytes from injury. Since both GC and Pio activate nuclear receptors (NR3C1 and PPARG, respectively) we hypothesized that proteinuria-reducing effects result from overlapping glomerular gene transcriptional patterns.

## Methods

We performed transcriptome analyses on glomeruli isolated from GC (immunosuppressive)- and Pio (non-immunosuppressive)-treated rats 11 days after NS induction with PAN (n=4/group).

## Results

Unsupervised clustering revealed partial reversibility of PAN-associated mRNA changes after either GC or Pio. IPA analyses + web-based bioinformatic platforms identified 29 genes-of-interest common to GC- and Pio-induced proteinuria reduction, which included ECM remodeling, lipid metabolism, DNA-binding, and cytoskeletal organization. Using real-time PCR expression differences, 29 genes-of-interest were selected for further analysis, which when clinically correlated with the *Nephroseq* human FSGS database suggested a mechanistic role for glomerular ECM regulation in NS proteinuria reduction. Also, glomerular cell deconvolution using published single-cell glomerular transcriptome profiles identified podocyte- and mesangial cell-specific perturbations in gene expression during NS and with treatments. Finally, validation of selected genes-of-interest using PAN-induced injury of human podocytes and mesangial cells confirmed significant upregulation of *LGALS3 (primary role in cell adhesion) and MMP2/ACTA2 (primary role in ECM degradation/cell motility or integrity) respectively.*

## Conclusion

These studies identified glomerular cell-specific transcripts common to both immunosuppressive and non-immunosuppressive proteinuria reduction that identify possible targets for future treatment for NS.

# Abstract: 1 The role of podocytes in blood pressure regulation: a multi-omic causality analysis

**Authors:** Dr James Eales, Dr Xiao Jiang, Dr Xiaoguang Xu, Dr Sushant Saluja, Dr Eddie Cano-Gamez, Dr Hui Guo, Dr Gosia Trynka, Prof Andrew Morris, Sir Nilesh Samani, Prof Fadi Charchar, Prof Maciej Tomaszewski

**Affiliations:** ^1^University of Manchester, Manchester, United Kingdom; ^2^Wellcome Sanger Institute, Cambridge, United Kingdom; ^3^University of Leicester, Leicester, United Kingdom; ^4^Federation University, Ballarat, Australia

## Background

Blood pressure (BP) is a complex polygenic trait, with over 800 common genetic variants identified by genome-wide association studies (GWAS). The mechanisms through which these variants operate remain largely elusive, this may be partly explained because the key tissues and cells of relevance to BP regulation have not yet been fully exploited in omics-style studies. Here we explore the causal links between podocyte gene expression and blood pressure.

## Methods

We examined the effects of 821 common BP GWAS variants on the human kidney transcriptome using a collection of 430 human kidneys from the Human Renal Tissue Resource. A podocyte-specific gene expression profile was derived from 41778 renal cells profiled by single cell RNA-sequencing. We applied an ensemble of Mendelian randomisation (MR) methods to determine which podocyte enriched genes show evidence of a causal link with blood pressure.

## Results

We identified 418 kidney genes modified in expression by BP associated genetic variants, of these 22 showed enriched expression in podocytes. Our Mendelian randomisation analysis identified 179 kidney genes showing evidence of a causal association with BP, five of those showed enriched expression in podocytes.

## Conclusions

Our set of 5 podocyte enriched genes (with a causal link to BP) include known glomerular genes, those with unknown function and those that highlight the limitations of single-cell transcriptomics in renal tissue.

# Abstract: 2 Podocyte-omics; sexually dimorphic biology and responses to mTOR inhibition

**Authors:** Ola Al Diab, Dr.rer.nat Dennis Gürgen, Dr.rer.nat Christin Sünkel, Dr. Eric Blanc, Prof. Dr. med Markus Rinschen, Dr.rer.nat Raphaela Fritsche, Dr. Florian Grahammer, Dr. Jennifer Kirwan, Prof. Dr. Nikolaus Rajewsky, Prof. Dr. med. Tobias B. Huber, Dr. med. Angelika Kusch

**Affiliations:** ^1^Charité Universitätsmedizin Berlin, Medizinische Klinik m. S. Nephrologie und internistische Intensivmedizin, Campus Virchow Klinikum, Berlin, Germany; ^2^Experimental Pharmacology & Oncology Berlin-Buch GmbH, Berlin-Buch, Germany; ^3^Celonic AG, Basel, Swaziland; ^4^BIH Core Unit Bioinformatics, Berlin Institute of Health at Charité (BIH), Berlin, Germany; ^5^Universitätsklinikum Hamburg - Eppendorf (UKE), III. Medizinische Klinik und Poliklinik, Hamburg, Germany; ^6^BIH Core Facility Metabolomics, Berlin Institute of Health at Charité (BIH), Berlin, Germany; ^7^Berlin Institute for Medical Systems Biology (BIMSBI), Berlin, Germany; ^8^Charité Universitätsmedizin Berlin, Medizinische Klinik m. S. Nephrologie und internistische Intensivmedizin, Campus Virchow Klinikum / Berlin Institute of Health at Charité (BIH), Berlin, Germany

*Sex differences in podocyte molecular signatures and homeostasis might explain clinically observed sex differences in kidney diseases and could provide a rationale for novel approaches in personalized therapies.*

Thus, male and female ROSA mT/mG-NHPS2Cre mice with GFP-expression restricted to podocytes were treated with rapamycin as a general metabolic stress model for three weeks to investigate intrinsic and stress-related sexual dimorphism of podocytes on a genomic and proteomic level. Metabolomics of renal cortex tissues were included to investigate functional consequences of rapamycin.

Kidney function and morphology remained normal in all experimental groups, yet RNA sequencing revealed strong intrinsic sex-differences with more than 1700 mRNA transcripts significantly sex- differently expressed. Furthermore, stress responses due to mTOR inhibition displayed sexual dimorphism in transcriptomes and metabolomes and signaling pathways as assessed by western blots. Gene set enrichment analyses pointed to sex differences in enriched GOs related to transcription, cytoskeleton, focal adhesion, mitochondria and translation. Additionally, proteomics displayed an increased abundance of cytoskeleton and focal adhesion molecules in females. Rapamycin treatment abolished the significant transcriptional sex differences, mainly due to increased changes in the male podocyte transcriptome. Metabolomics further supported the finding that females were less susceptible towards stress induced by mTOR inhibition showing less accumulation of amino acids and glycolysis metabolites after rapamycin.

In conclusion, for the first time podocyte-specific sexual dimorphism was identified in gene regulation and protein expression patterns in addition to sex-different metabolic changes in stress responses, which might explain and contribute significantly to sex differences in renal disease susceptibilities and progression.

# Abstract: 3 Applying proteomics to investigate basement membrane assembly in human kidney organoids

**Authors:** Dr. Mychel Morais, Dr. Pinyuan Tian, Dr. Craig Lawless, Miss Louise Hopkinson, Dr Steven Woods, Dr. Telma Zorn, Dr. Susan Kimber, Dr. Roy Zent, Dr. Rachel Lennon

**Affiliations:** ^1^The University of Manchester, Manchester, United Kingdom; ^2^University of Sao Paulo, Sao Paulo, Brazil; ^3^Vanderbilt University Medical Center, Nashville, USA

## Background

Basement membranes (BM) are dense, condensed sheets of extracellular matrix that underlie continuous layers of cells, and essential components include type IV collagens, laminins, nidogens and heparan-sulfate proteoglycans, plus many other minor static and mobile components. BMs are assembled early during embryogenesis and undergo a highly dynamic remodeling; recent studies in *Drosophila* and *C. elegans* development have proposed conserved hierarchical sequences for BM assembly and repair. Here, we investigated the temporal assembly and remodeling of BM in human iPSC-derived kidney organoids, a valuable tool to model nephrogenesis in vitro.

## Methods

We recently characterized a BM gene network comprising 222 human genes (encoding 160 BM proteins and 62 cell surface interactors) and combined it with label-free quantitative mass- spectrometry.

## Results

We identify 78 BM proteins expressed by kidney organoids during differentiation in 3D culture. Overall, we observed increasing levels of BM proteins along the differentiation time-course, and distinct pattern of temporal expression for key BM proteins as such early components (COL4A1, COL4A2, LAMB1, LAMC1) and glomerular BM laminins (LAMA5, LAMB2), altogether indicating gradual BM assembly and remodeling over time. We also detected increased levels of EPB41L5 (implicated in incorporating laminin-511 and −521 into specialized kidney BMs) concomitant with the upregulation of deposited laminin alpha-5, suggesting a recapitulation of BM specialization in vitro. Integrated proteomic analyses between kidney organoid, human and mouse kidney datasets indicated that kidney organoids recapitulate a kidney BM signature seen *in vivo.*

## Conclusion

we demonstrated that kidney organoids are a high-fidelity system to study BM assembly in vertebrates.

# Abstract: 1 Podocyte cell cycle activation during chronic kidney disease progression

**Authors:** Dr Camille Nicolas Frank, Dr Xiaogang Hou, Mr Rui Zhao, Mr Joshua R Hansen, Dr Geremy Clair, Mr Fadi Salem, Dr Roger E De Filippo, Dr Paolo Cravedi, Dr Kevin V Lemley, Dr Laura Perin

**Affiliations:** ^1^GOFARR Laboratory for Organ Regenerative Research and Cell Therapeutics in Urology, Saban Research Institute, Division of Urology, Children's Hospital Los Angeles, Los Angeles, USA; ^2^Division of Nephrology, Department of Pediatrics, Boston Children's Hospital, Harvard Medical School, Boston, USA; ^3^Biological Science Division, Integrative Omics, Pacific Northwest National Laboratory, Richland, USA; ^4^Division of Nephrology, Icahn School of Medicine at Mount Sinai, New York, USA; ^5^Division of Nephrology, Department of Pediatrics, Children's Hospital Los Angeles, Keck School of Medicine, University of Southern California, Los Angeles, USA

## Background

Progression of glomerulosclerosis is associated with loss of podocytes and subsequent glomerular tuft instability. A decreased number of podocytes may be able to preserve tuft stability through cell hypertrophy associated with cell cycle re-entry. At the same time, re-entry into the cell cycle can lead to podocyte detachment from the glomerular basement membrane, if podocytes cross the G1/S checkpoint and undergo abortive cytokinesis. Methods: To study cell cycle dynamics during CKD development, we used a FUCCI mouse model (fluorescence ubiquitination-based cell cycle indicator) affected by X-linked Alport Syndrome (AS). This model has progressive CKD and expresses cell cycle fluorescent reporters exclusively in podocytes. We quantified podocytes cell cycle distribution in WT and AS mice at different ages and collected podocytes in GO and G1 for proteomics studies. Results: We showed that with development of CKD, an increasing fraction of podocytes *in vivo* are in G1 or later cell cycle stages. G1 and G2 podocytes are hypertrophic. Heterozygous female mice, with milder manifestations of CKD, show G1 fraction numbers intermediate between WT and male AS mice. Proteomic analysis showed differences in cytoskeleton re-organization and metabolic processes between podocytes in GO and G1 in disease vs. WT and indicate alteration of specific proteins also identified in human AS podocytes. Conclusion: our data showed that, during progressive CKD, the podocyte cell cycle distribution changes dramatically, suggesting that cell cycle manipulation may have a role in treatment of various progressive glomerular diseases characterized by podocytopenia.

# Abstract: 2 Prevalence and risk factors for the development of sickle cell nephropathy in children

**Authors:** Ms. Oyindamola Adebayo, Dr. DieuMerci Betukumesu, Dr. Agathe Nkoy, Ms. Oluyomi Adesoji, Dr. Pepe Ekulu, Prof. Dr. Lambertus van den Heuvel, Prof. Dr. Elena Levtchenko, Prof. Dr. Veerle Labarque

**Affiliations:** ^1^Centre for Molecular and Vascular Biology, Department of Cardiovascular Sciences, Katholieke Universiteit Leuven, Leuven, Belgium; ^2^Department of Development and Regeneration, Katholieke Universiteit Leuven, Leuven, Belgium; ^3^Division of Nephrology, Department of Paediatrics, University Hospital of Kinshasa, Faculty of Medicine, University of Kinshasa, Kinshasa, Congo, the Democratic Republic of the; ^4^Cologne Centre for Genomics, University of Cologne, Cologne, Germany; ^5^Department of Paediatric Nephrology, Radboud University Medical Centre, Nijmegen, Netherlands; ^6^Department of Paediatric Nephrology University Hospital Leuven, Leuven, Belgium; ^7^Department of Paediatric Haematology University Hospital Leuven, Leuven, Belgium

## Background

Clinical and genetic factors have been reported to influence the development of sickle cell nephropathy (SCN). However, such data remain limited in the paediatric population. Our study aimed to determine the prevalence of markers of kidney damage and to examine the association between these markers and clinical and genetic factors in children with sickle cell anaemia (SCA).

## Methods

In a cross-sectional study, we enrolled 361 children with sickle cell disease from the Democratic Republic of Congo. Participants were genotyped for β-globin gene, Apolipoprotein L1 *(APOL1)* risk variants, and Haem oxygenase-1 *(HMOX1)* GT dinucleotide repeats. As outcomes, albuminuria was defined as urinary albumin-to-creatinine ratio (ACR) ≥30mg/g, decreased estimated GFR with creatinine (eGFRcr) when <60 ml/min/1.73m^2^ and hyperfiltration when eGFRcr >130 and 140 ml/min/1.73m^2^ for female and male, respectively. Results: Genetic sequencing confirmed 326 participants as SCA patients. Albuminuria, hyperfiltration and decreased eGFRcr were present in 65 (20%), 93 (28%) and 18 (5.50%) patients, respectively. Regression analysis revealed frequent blood transfusions (P = 0.01), lower diastolic blood pressure (P = 0.04) and male gender (P = 0.02) as clinical parameters of SCN. *APOL1* high risk genotype (G1/G1, G2/G2 and G1/G2) was significantly associated with albuminuria (P = 0.04) and hyperfiltration (P = 0.001). *HMOX1* GT dinucleotide long repeats were significantly associated with lower eGFRcr (P = 0.03).

## Conclusions

The study revealed a high burden of kidney damage among Congolese children, and evidence has been provided of possible role of *APOL1* and *HMOX1* in making individuals more susceptible to kidney complications.

# Abstract: 3 Generation of novel 3D co-culture systems to study podocytopathies ex vivo in a personalized manner

**Authors:** Victoria Rose, Dr. rer. nat. Nina Sopel, Alexandra Ohs, Dr. med. vet. Christina Warnecke, Prof. Dr. med. Mario Schiffer, Prof. Dr. med. Janina Müller-Deile

**Affiliations:** Universitätsklinikum Erlangen - Department of Medicine 4 - Nephrology and Hypertension, Erlangen, Germany

## Background

Marker expression, morphology and extracellular matrix production is known to be altered and cell-cell- interaction is limited in classical 2D cell culture. To improve culture conditions and to investigate cell- cell-interaction and communication, we aim to generate a 3D glomerular co-culture model. Moreover, we want to study podocyte disease by personalizing the 3D co-culture model with patient-derived podocytes.

## Methods

Glomerular spheroids formed by co-culture of immortalized human podocytes, glomerular endothelial cells and mesangial cells in a hanging droplet or microwells. Spheroid formation was imaged in time laps experiments, on histological sections, and by qPCR. Patient-specific podocytes were differentiated from hiPSC, which were initially generated by reprogramming skin fibroblasts.

## Results

Glomerular spheroids showed a capsule of CD31 positive endothelial cells. Moreover, extracellular matrix could be imaged by SEM and qPCR showed an increased expression of collagen IV and laminin in 3D culture compared to classical 2D culture. TEM revealed cell protrusions of podocytes that were not seen in monocultures.

Reprogramming of skin fibroblasts led to changes in phenotype with a high nuclei to body ratio, which expressed pluripotency markers. Differentiation to hiPSC-podocytes resulted in the development of primary and secondary foot processes and expression of podocyte-specific markers that were partially not found in our immortalized podocytes.

## Conclusion

3D co-culture provides better physiological conditions for glomerular cells.

Furthermore, iPSC-derived podocytes show better cell type specific characteristics than immortalized podocyte and enables to generate patient-derived podocytes with specific mutations that will allow to study cell-cell interaction and communication in podocyte disease.
